# Capturing functional connectomics using Riemannian partial least squares

**DOI:** 10.1038/s41598-023-44687-2

**Published:** 2023-10-13

**Authors:** Matthew Ryan, Gary Glonek, Jono Tuke, Melissa Humphries

**Affiliations:** School of Computer and Mathematical Sciences, The University of Adelaide, Adelaide, 5005 Australia

**Keywords:** Autism spectrum disorders, Applied mathematics, Statistics

## Abstract

For neurological disorders and diseases, functional and anatomical connectomes of the human brain can be used to better inform targeted interventions and treatment strategies. Functional magnetic resonance imaging (fMRI) is a non-invasive neuroimaging technique that captures spatio-temporal brain function through change in blood-oxygen-level-dependent (BOLD) signals over time. FMRI can be used to study the functional connectome through the functional connectivity matrix; that is, Pearson’s correlation matrix between time series from the regions of interest of an fMRI image. One approach to analysing functional connectivity is using partial least squares (PLS), a multivariate regression technique designed for high-dimensional predictor data. However, analysing functional connectivity with PLS ignores a key property of the functional connectivity matrix; namely, these matrices are positive definite. To account for this, we introduce a generalisation of PLS to Riemannian manifolds, called R-PLS, and apply it to symmetric positive definite matrices with the affine invariant geometry. We apply R-PLS to two functional imaging datasets: COBRE, which investigates functional differences between schizophrenic patients and healthy controls, and; ABIDE, which compares people with autism spectrum disorder and neurotypical controls. Using the variable importance in the projection statistic on the results of R-PLS, we identify key functional connections in each dataset that are well represented in the literature. Given the generality of R-PLS, this method has the potential to investigate new functional connectomes in the brain, and with future application to structural data can open up further avenues of research in multi-modal imaging analysis.

## Introduction

The functional and anatomical connections of the human brain form complex networks that link the infrastructure of our minds. Understanding these connectomes has the potential to provide insight into the effect of neurological diseases which can be used to better inform targeted interventions and treatment strategies^1,2^. In particular, the functional connectome can shed new light onto psychiatric and neurological conditions such as schizophrenia and autism spectrum disorder (ASD), two conditions that alter brain function from healthy, neurotypical controls^3,4^.

A popular approach used to investigate brain function is functional magnetic resonance imaging (fMRI), a non-invasive neuroimaging technique that measures the blood-oxygenation-level-dependent (BOLD) signal over time as a correlate of brain activity^5^. An fMRI image is a complex spatio-temporal picture of the brain with voxels (volumetric pixels) describing the spatial location and a time series for each voxel describing the BOLD signal. To reduce the spatial complexity, voxels can be collated into user-specified regions of interest (ROIs). Functional connectomes can then be investigated through Pearson’s correlation matrix between ROIs, known as the functional connectivity matrix.

One approach to investigating functional connectivity is using the partial least squares (PLS) regression method. Introduced by Wold^6^ for use in chemometrics, PLS is an extension of multivariate multiple regression to high-dimensional data that predicts the response data from a set of lower-dimensional latent variables (that is, unobserved variables) constructed from the predictor data. Popularised for fMRI by McIntosh et. al.^7^, PLS has been used to explore the relationships between fMRI data and either behavioural data, experimental designs, or seed region activation^8^. However, standard PLS ignores the structure of functional connectivity data—functional connectivity matrices are correlation matrices and hence positive definite, that is, they have non-negative eigenvalues. By ignoring the positive definite criteria, standard PLS on functional connectivity data has the potential to ignore intricate non-linearities in the data and provide invalid predictions from the model.

For a given number of ROIs *R*, the space of $R\times R$ symmetric positive definite matrices—which includes functional connectivity matrices—forms a convex cone in $R(R+1)/2$-dimensional Euclidean space, that is, they form a shape much like an ice-cream cone in high dimensional Euclidean space that has a singularity at the origin. However, when considered with the affine invariant geometry^9^, the space of symmetric positive definite matrices becomes a complete Riemannian manifold—a general mathematical space where we can perform calculus—with non-positive curvature, that is, it curves in on itself in interesting and complex ways. By considering this non-linear geometry on symmetric positive definite matrices we can glean interesting new insights into functional connectivity (see Pennec et. al.^10^ and citations therein).

The process of generalising statistical models to Riemannian manifolds has the potential to investigate non-linearities in the data^11^, and has been gaining significant interest in neuroimaging in recent years^10^. Fletcher^12^ proposed principal geodesic analysis as a generalisation of principal components analysis to Riemannian manifolds for applications in shape analysis for medically-defined anatomical shapes. Later, Fletcher^11^ generalised simple linear regression to Riemannian manifolds, called geodesic regression, to investigate how medically-defined anatomical shapes can change with age. Following this vein of research, Kim^13^ generalised canonical correlations analysis to Riemannian manifolds to explore multi-modal imaging relationships between diffusion tensor images and structural MRI in Alzheimer’s patients. However, PLS, which is closely related to all of these methods, has not yet been generalised to Riemannian manifolds.

Here we propose an extension of the PLS model to allow Riemannian manifold response and predictor data, which we call Riemannian partial least squares (R-PLS). The R-PLS model then allows us to predict from functional connectivity data while accounting for the intricate relationships enforced by the positive definite criteria. To fit the R-PLS model, we propose the tangent non-linear iterative partial least squares (tNIPALS) algorithm, which is related to previously proposed applications of PLS for functional connectivity data in the literature^14–17^. We determine the optimal number of latent variables using cross validation. To aid in interpretability of the high-dimensional functional connectivity data, we determine significant functional connections identified by R-PLS using permutation tests on the variable importance in the projection (VIP) statistic^18^, a popular measure of variable importance from standard PLS.

We apply R-PLS to two datasets and two different ROI atlases to demonstrate its versatility in predicting phenotype data from functional connectivity. First is the COBRE dataset^19^ which investigates differences in functional connectivity between healthy controls ($n=74$) and patients with schizophrenia ($n=72$). We consider two separate atlases on the COBRE dataset to test the generalisability of R-PLS across atlases; the multi-subject dictionary learning (MSDL) atlas^20^ to look at a low-dimensional (39 ROIs), data-driven atlas, as well as the automated anatomic labelling (AAL) atlas, a higher-dimensional (116 ROIs) anatomical atlas. The second dataset is the ABIDE dataset from the New York University imaging site^21^ which investigates differences in functional connectivity between typical healthy controls ($n=98$) and subjects with ASD ($n=75$). We consider the ABIDE data in the AAL atlas^22^ to investigate the generalisability of R-PLS across datasets. Thus, when predicting using the MSDL atlas we are considering 780 unique functional connections (since $R=39$), and when predicting using the AAL atlas we are considering 6786 unique functional connections (since $R = 116$).

## Results

For each dataset and atlas we predict the multivariate phenotype information (age and group for the COBRE dataset, as well as sex and eye status for ABIDE) from the functional connectivity data using the R-PLS model. The categorical variables group, sex, and eye status were represented by binary values, and all phenotype information was standardised to have mean zero and standard deviation one. When analysing functional connectivity matrices in the AAL atlas there was one matrix in the COBRE dataset and 24 matrices in the ABIDE dataset which had low-rank, and hence were not positive definite. To deal with these low-rank functional connectivity matrices, we consider regularised functional connectivity matrices ${{\tilde{F}}} = {F} + {I}$ following Venkatesh et. al.^23^, where *I* is the $116\times 116$ identity matrix. We compare R-PLS to the standard PLS model using the upper triangle of the functional connectivity matrices as the predictors (raw correlations), as well as their Fisher transformed values (Fisher correlations).

### Model fitting

We determine the optimal number of latent variables *K* in the PLS model through ten-fold cross validation using the “within one standard error” rule^24^ when minimising the root mean square error (RMSE) on the multivariate phenotype information. Due to the interest in the COBRE and ABIDE datasets in investigating the differences between healthy controls and patients, we also present the group classification metrics of accuracy, sensitivity, specificity, and area under the operator receiver curve (AUC). Since we have represented group as a binary value, we classify subjects in the patient group (schizophrenia or ASD) if their predicted group score is greater than zero, and in the control group otherwise. Graphs of the cross validation results can be found in the supplementary material (Fig. S1).

For the COBRE dataset with the MSDL atlas, ten-fold cross validation showed that $K = 2$ latent variables was the most parsimonious, within one standard error of the minimum RMSE ($K=3$). When compared with Euclidean PLS using raw and Fisher-transformed correlations, R-PLS outperformed both methods across all metrics except for specificity in group prediction (Table 1) . However, all three methods produced similar results for every metric.

**Table 1:** Mean (SE) 10-fold cross validation results for Riemannian partial least squares (R-PLS) on the COBRE and ABIDE datasets, and Euclidean PLS using the raw and Fisher transformed correlations.

	Riemannian	Raw correlations	Fisher correlations
COBRE-MSDL			
K	2	3	3
Full model metrics (SE)			
$R^2$	**0.25 (0.035)**	0.23 (0.033)	0.23 (0.036)
RMSE	**1.20 (0.036)**	1.21 (0.025)	1.21 (0.026)
Group classification (SE)			
Accuracy	**0.75 (0.045)**	0.73 (0.032)	0.74 (0.032)
Sensitivity	**0.81 (0.035)**	0.70 (0.057)	0.72 (0.055)
Specificity	0.69 (0.071)	**0.76 (0.048)**	**0.76 (0.048)**
AUC	**0.81 (0.039)**	0.78 (0.027)	0.79 (0.024)
COBRE-AAL			
K	3	3	3
Full model metrics (SE)			
$R^2$	**0.43 (0.034)**	0.38 (0.043)	0.38 (0.043)
RMSE	**1.04 (0.042)**	1.08 (0.047)	1.08 (0.047)
Group classification (SE)			
Accuracy	**0.79 (0.034)**	0.76 (0.038)	0.76 (0.038)
Sensitivity	**0.80 (0.049)**	0.75 (0.063)	0.75 (0.063)
Specificity	**0.78 (0.030)**	0.76 (0.030)	0.76 (0.030)
AUC	**0.86 (0.031)**	0.83 (0.039)	0.83 (0.040)
ABIDE			
K	3	3	3
Full model metrics (SE)			
$R^2$	**0.15 (0.015)**	0.07 (0.016)	0.07 (0.016)
RMSE	**1.80 (0.051)**	1.89 (0.059)	1.89 (0.059)
Group classification (SE)			
Accuracy	**0.58 (0.027)**	0.55 (0.032)	0.54 (0.032)
Sensitivity	**0.61 (0.058)**	0.52 (0.064)	0.51 (0.063)
Specificity	0.53 (0.063)	**0.58 (0.065)**	**0.58 (0.065)**
AUC	**0.64 (0.016)**	0.61 (0.047)	0.60 (0.046)

The value *K* represents the optimal number of latent variables for each model when minimising the root mean square error (RMSE) using the within one standard error rule. The full model metrics are the multivariate $R^2$ and RMSE. The group classification metrics of accuracy, sensitivity, specificity, and area under the operator receiver curve (AUC) look at the classification for subject group only. R-PLS is the best model for both datasets and atlases over all model metrics, except for specificity (bold values).

When considering the COBRE dataset in the AAL atlas, ten-fold cross validation showed that $K = 3$ latent variables was the most parsimonious, within one standard error of the minimum RMSE ($K=3$). Similar to the results from the MSDL atlas, we found that R-PLS outperformed the Euclidean PLS methods across all metrics, although now with the inclusion of group specificity (Table 1). When using the AAL atlas on the COBRE dataset, we observe a substantial increase in the cross validated $R^2$ value over the Euclidean methods.

For the ABIDE dataset, ten-fold cross validation found $K = 3$ latent variables was the most parsimonious, within one standard error of the minimum RMSE ($K=6$). When compared with Euclidean PLS using the raw and Fisher-transformed correlations, R-PLS outperformed both methods across all metrics except for specificity in group classification (Table 1). In particular, the $R^2$ value and AUC for R-PLS was substantially larger than the Euclidean methods.

### Interpretation

To investigate the functional connectomes associated to each phenotype variable, we consider the regression coefficient matrix ${\beta }_{PLS}$ (see Eq. 4 in the “Methods” section) where the $i^{th}$ column represents the effect of the functional connectivity matrix on the $i^{th}$ response variable (age, group, sex, or eye status). Much like the regression coefficients in ordinary least squares, the coefficient matrix $\beta _{PLS}$ captures the multivariate association between functional connectivity and the phenotype data. We determine which functional connections are significantly associated with the phenotype variables through a permutation test of the VIP statistic (Eq. 5 in the “Methods” section) using 200 permutations at a significance level of $\alpha =0.05$, as described in the “Methods” section. All analysis was performed using r^25^.

We visualise the columns of the matrix ${\beta }_{PLS}$ as symmetric matrices in the tangent space of the Fréchet mean for each dataset, and represent them as connectomes on standard brains images using the nilearn package in python. To assist in visualising patterns in the regression coefficients across the connectome, we average the coefficient values across all connections within and between predefined resting state networks similar to Wong et. al.^14^. The within-network connectivity is then the average coefficient of all connections within a single resting state network, and the between-network connectivity is the average coefficient of all connections between two resting state networks. For the MSDL atlas this involves reducing the 39 ROIs to the 17 resting state networks associated to the atlas^26^. For the AAL atlas, we associate the 116 ROIs to the seven resting-state networks suggested by Parente and Colosimo^27^ and an eighth containing the cerebellum and vermis, which we call the cerebellum network. The resting state networks for the MSDL and AAL atlases are visualised in the supplementary material (MSDL in Figs. S2–S7, AAL in Figs. S8–S10).

**Figure 1:**
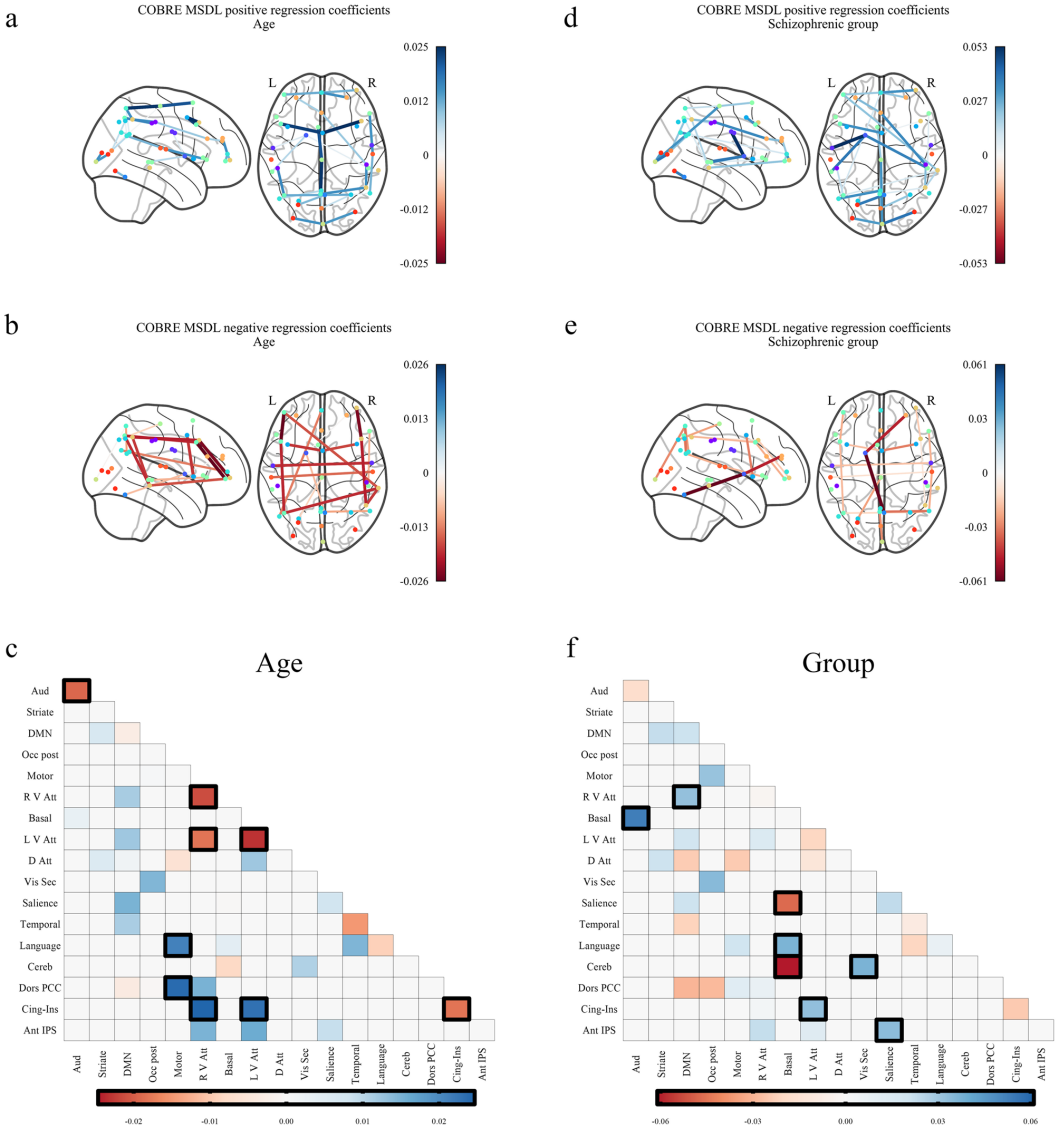
Significant regression coefficients for predicting age and schizophrenia as measured by variable importance in the projection (VIP) for the Riemannian partial least squares (R-PLS) model on the COBRE dataset and the multi-subject dictionary learning (MSDL) atlas with $K = 2$ latent variables, visualised as connectomes and symmetric matrices. Blue values represent connections that are positively associated with the phenotype, that is, an increase in connectivity between two regions with a blue edge would indicate an increase in the phenotype variable. Conversely, red values are connections that are negatively associated with the phenotype, that is, an increase in connectivity between two regions with a red edge would indicate a decrease in the phenotype variable. (**a**) Shows the connections that increase with age, (**b**) shows the connections that decrease with age, and (**c**) shows the average coefficient values for age between the 17 resting state networks of the MSDL atlas^26^ (Figs. S2–S7). (**d**) Shows the connections that increase for patients with schizophrenia, (**e**) shows the connections that decrease for patients with schizophrenia, and (**f**) shows the average coefficient values for schizophrenia between the 17 resting state networks of the MSDL atlas^26^ (Figs. S2–S7). The darker outlined boxes in (**c**) and (**f**) show the top $25\%$ influential regions as measured by the absolute coefficient value within and between each network. The network abbreviations in (**c**) and (**f**) are: *Aud* auditory, *Striate* striate, *DMN* default model network, *Occ Post* occipital posterior, *Motor* motor network, *R V Att* right ventral attention network, *Basal* Basal Ganglia, *L V Att* left ventral attention network, *D Att* dorsal attention network, *Vis Sec* secondary visual cortex, *Salience* salience network, *Temporal* temporal network, *Language* language network, *Cereb* cerebellum, *Dors PCC* dorsal posterior cingulate cortex, *Cing-Ins* cingulate-insula network, *Ant IPS* anterior intraparietal sulcus.

**Figure 2:**
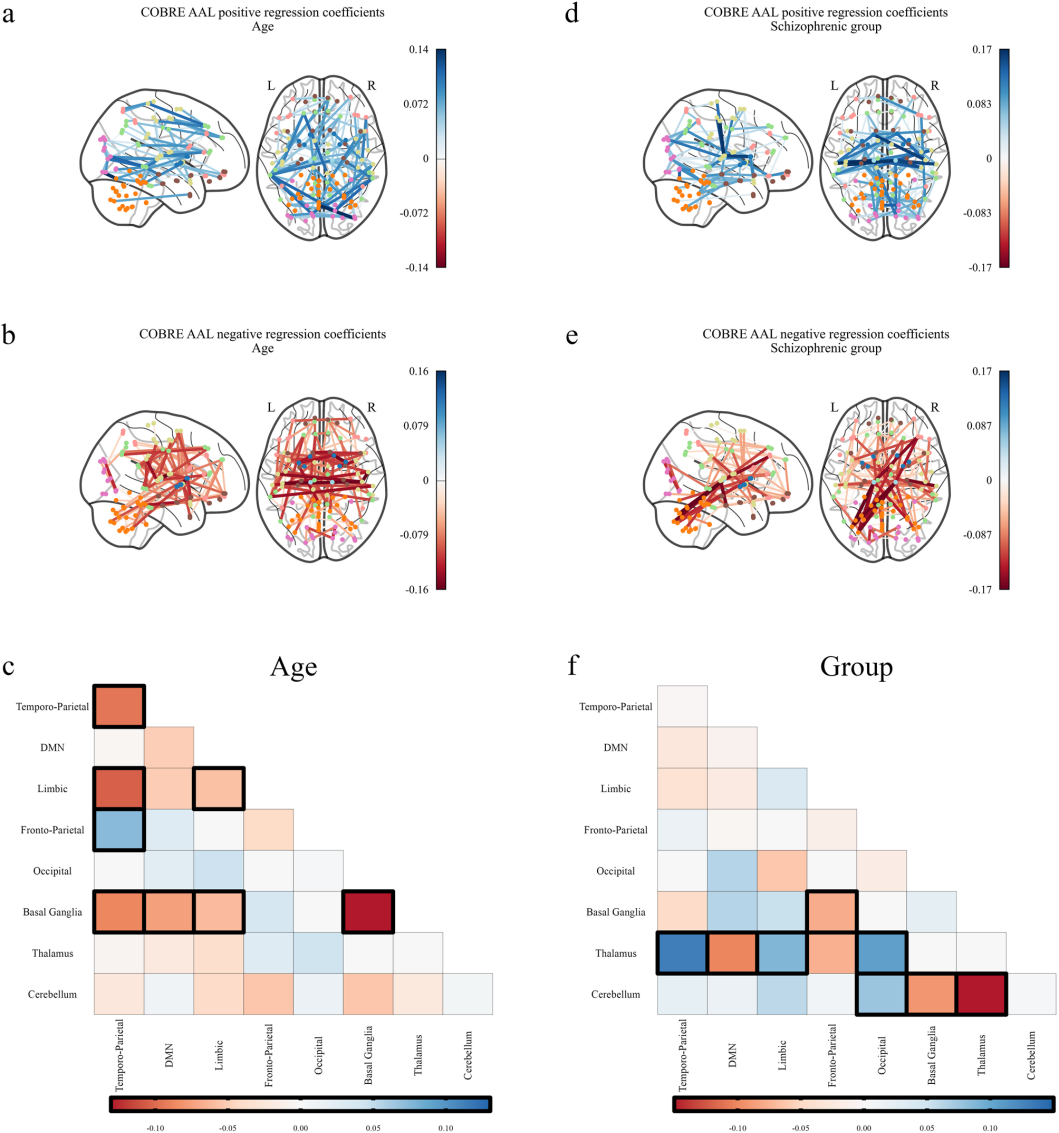
Significant regression coefficients for predicting age and schizophrenia as measured by variable importance in the projection (VIP) for the Riemannian partial least squares (R-PLS) model on the COBRE dataset and the automated anatomic labelling (AAL) atlas with $K = 3$ latent variables, visualised as connectomes and symmetric matrices. Blue values represent connections that are positively associated with the phenotype, that is, an increase in connectivity between two regions with a blue edge would indicate an increase in the phenotype variable. Conversely, red values are connections that are negatively associated with the phenotype, that is, an increase in connectivity between two regions with a red edge would indicate a decrease in the phenotype variable. (**a**) Shows the connections that increase with age, (**b**) shows the connections that decrease with age, and (**c**) shows the average coefficient values for age between the 7 resting state networks identified by Parente and Colosimo^27^ and the cerebellum (Figs. S8–S10). (**d**) Shows the connections that increase for patients with schizophrenia, (**e**) shows the connections that decrease for patients with schizophrenia, and (**f**) shows the average coefficient values for schizophrenia between the 7 resting state networks identified by Parente and Colosimo^27^ and the cerebellum (Figs. S8–S10). The darker outlined boxes in (**c**) and (**f**) show the top $25\%$ influential regions as measured by the absolute coefficient value within and between each network. In (**c**) and (**f**), *DMN* default mode network.

**Figure 3:**
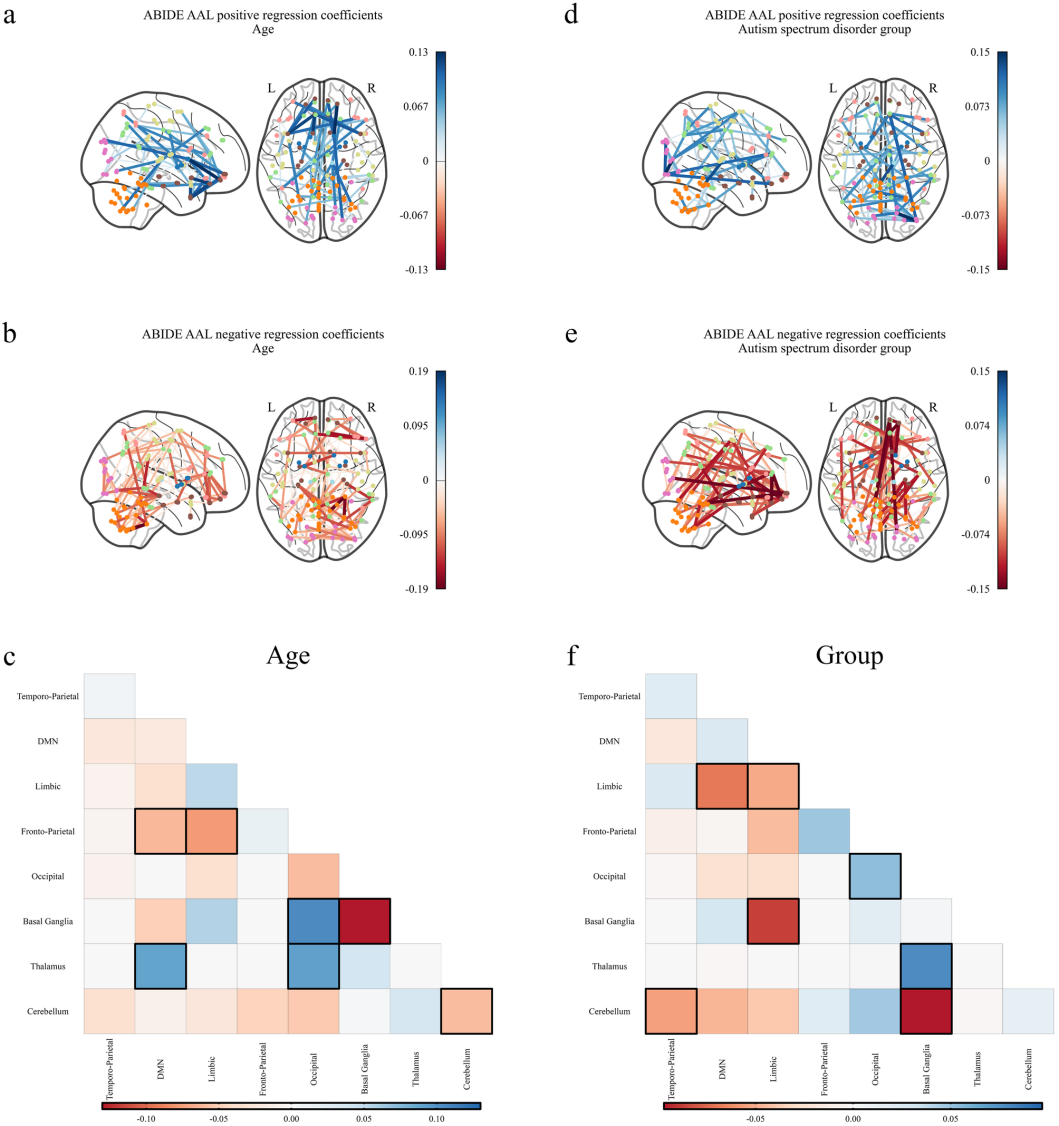
Significant regression coefficients for predicting age and autism spectrum disorder (ASD) as measured by variable importance in the projection (VIP) for the Riemannian partial least squares (R-PLS) model on the ABIDE dataset and the automated anatomic labelling (AAL) atlas with $K = 3$ latent variables, visualised as connectomes and symmetric matrices. Blue values represent connections that are positively associated with the phenotype, that is, an increase in connectivity between two regions with a blue edge would indicate an increase in the phenotype variable. Conversely, red values are connections that are negatively associated with the phenotype, that is, an increase in connectivity between two regions with a red edge would indicate a decrease in the phenotype variable. (**a**) Shows the connections that increase with age, (**b**) shows the connections that decrease with age, and (**c**) shows the average coefficient values for age between the 7 resting state networks identified by Parente and Colosimo^27^ and the cerebellum (Figs. S8–S10). (**d**) Shows the connections that increase for patients with ASD, (**e**) shows the connections that decrease for patients with ASD, and (**f**) shows the average coefficient values for ASD between the 7 resting state networks identified by Parente and Colosimo^27^ and the cerebellum (Figs. S8–S10). The darker outlined boxes in (**c**) and (**f**) show the top $25\%$ influential regions as measured by the absolute coefficient value within and between each network. In (**c**) and (**f**), *DMN* default mode network.

**Figure 4:**
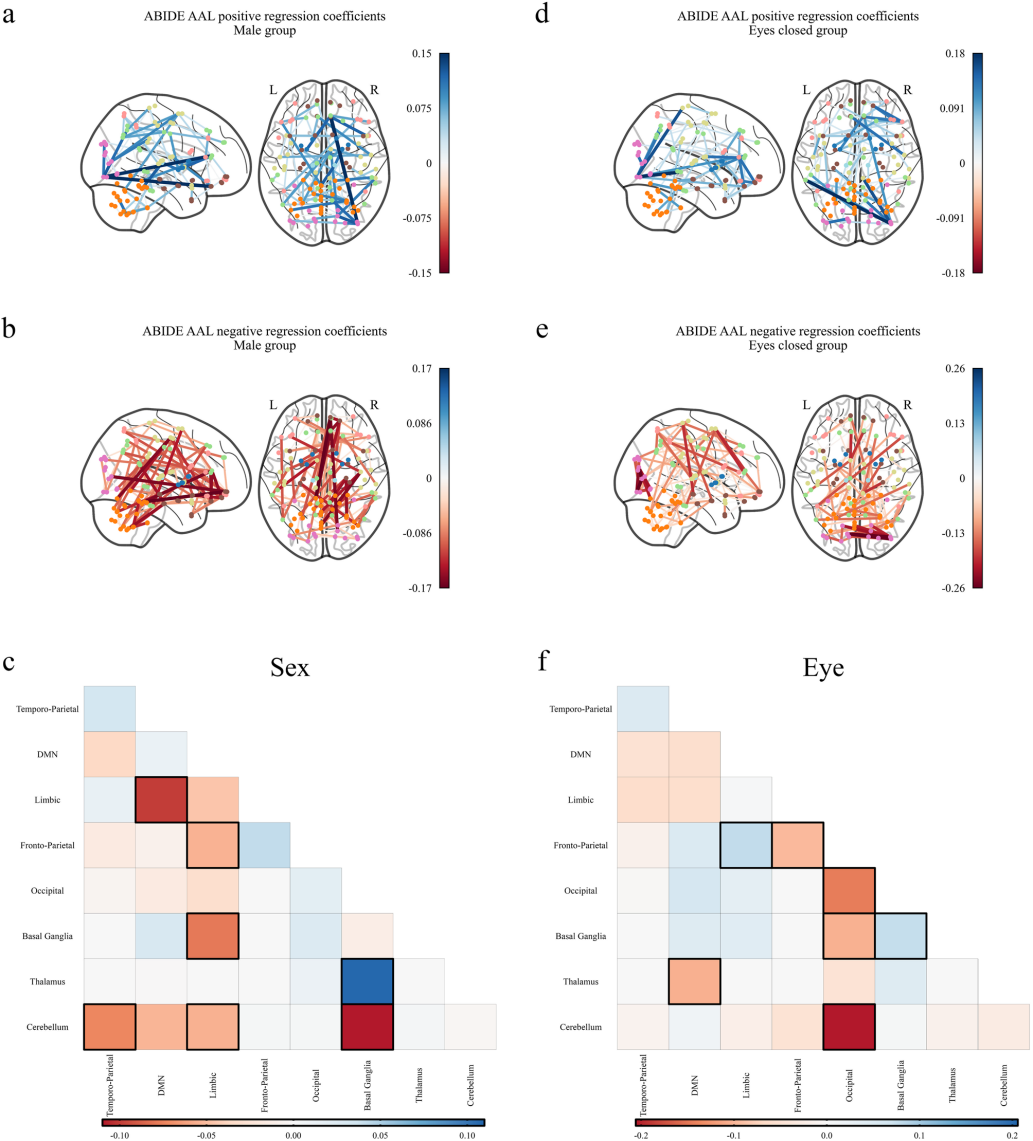
Significant regression coefficients for predicting sex and eye status as measured by variable importance in the projection (VIP) for the Riemannian partial least squares (R-PLS) model on the ABIDE dataset and the automated anatomic labelling (AAL) atlas with $K = 3$ latent variables, visualised as connectomes and symmetric matrices. Blue values represent connections that are positively associated with the phenotype, that is, an increase in connectivity between two regions with a blue edge would indicate an increase in the phenotype variable. Conversely, red values are connections that are negatively associated with the phenotype, that is, an increase in connectivity between two regions with a red edge would indicate a decrease in the phenotype variable. (**a**) Shows the connections that increase for males, (**b**) shows the connections that decrease for males, and (**c**) shows the average coefficient values for males between the 7 resting state networks identified by Parente and Colosimo^27^ and the cerebellum (Figs. S8–S10). (**d**) Shows the connections that increase for patients with eyes open, (**e**) shows the connections that decrease for patients with eyes open, and (**f**) shows the average coefficient values for the eyes open group between the 7 resting state networks identified by Parente and Colosimo^27^ and the cerebellum (Figs. S8–S10). The darker outlined boxes in (**c**) and (**f**) show the top $25\%$ influential regions as measured by the absolute coefficient value within and between each network. In (**c**) and (**f**), *DMN* default mode network.

For the COBRE dataset with the MSDL atlas, a permutation test of the VIP statistic with 200 permutations found 45 significant functional connections between ROIs as being predictive of age and subject group (Fig. 1). When considered with the AAL atlas, a permutation test of the VIP statistic with 200 permutations found 249 significant functional connections between ROIs as being predictive of age and subject group (Fig. 2). For the ABIDE dataset, a permutation test of the VIP statistic with 200 permutations found 196 significant functional connections between ROIs as being predictive of age, subject group, sex and eye status (Figs. 3 and 4).

Across both atlases for the COBRE dataset, an increase in subject age tended towards a decrease of within-network connectivity (as measured by a mean decrease in functional connectivity within-networks) with particular emphasis on the auditory network, cingulate insula, and left and right ventral attention networks in the MSDL atlas, and the temporo-parietal, limbic, and basal ganglia networks in the AAL atlas (Figs. 1, 2a–c). Increased age was associated with an increase in between-network connectivity, particularly for the MSDL atlas which shows increased connectivity involving the cingulate insula and the motor network. Notably, an increase in age is associated with a decrease in between-network connectivity for the basal ganglia in the AAL atlas but not in the MSDL atlas. In the ABIDE dataset, increased age was associated to both increased and decreased functional connectivity within resting-state networks (Fig. 3a–c). Although we observed increased between-network connectivity for the thalamus and occipital networks, the cerebellum and default mode network exhibited decreased between-network connectivity with age. Note that the decreased within-network connectivity for the basal ganglia with age is also present in the ABIDE dataset.

For subjects in the schizophrenic group, the basal ganglia exhibited both increased and decreased connectivity with other networks across both atlases (Figs. 1 and 2d–f). In particular, in the MSDL atlas there was a decrease in connectivity between the basal ganglia and the cerebellum and salience networks, whereas we observed an increase in connectivity between the basal ganglia and auditory and language networks for the schizophrenic group. The AAL atlas similarly demonstrates the reduced connectivity between the basal ganglia and the cerebellum, and further shows reduced connectivity between the basal ganglia and the fronto-parietal network. In contrast to the analysis with the MSDL atlas, using the AAL atlas suggest there is an increase in connectivity between the basal ganglia and the default mode network for schizophrenic subjects. For both atlases, the default mode network was highly discriminatory for the schizophrenic group showing both increased and decreased between-network connectivity. Finally, we note the the AAL atlas highlights connectivity with the thalamus as being highly discriminatory for the schizophrenic group, which is not represented in the MSDL atlas since there is no thalamus ROI.

For subjects with ASD we observed increased within-network connectivity with the exception of the limbic network (Fig. 3d–f). We also observed decreased between-network connectivity particularly for connections involving the cerebellum or the limbic networks. We observed similar connectivity patterns for subject sex (Fig. 4a–c).

For subjects with their eyes closed, our model suggests there was decreased within-network connectivity (Fig. 4d–f). With the exception of the default mode network, the limbic network, and the basal ganglia, we saw decreased between-network connectivity with particular emphasis on the occipital network.

## Discussion

The R-PLS model has identified many functional connections associated to age, ASD, schizophrenia, sex, and eye status that are well represented in the literature. Across both datasets and atlases, we identified the reduction of within-network connectivity with age that has been previously observed^28–30^, with exceptions in the temporo-parietal, fronto-parietal, and limbic networks in the ABIDE dataset and the salience network in the COBRE dataset, which all show an increase in connectivity with age. Further, both datasets exhibit the decreased connectivity within the default mode network, consistent with existing literature^31,32^. We also note that the previously observed decrease of within-network connectivity for the basal ganglia^33^ was prominent in the AAL atlas, but not the MSDL atlas. This is because there is only one region of interest for the basal ganglia in the MSDL atlas, so within-network connectivity is not defined.

For subjects with ASD, the decreased connectivity with the cerebellum^34^ and the limbic^35^ networks have been previously observed. However, the decreased between-network connectivity suggested by R-PLS is in contradiction with existing literature^14,36^; in particular, Wong et. al.^14^ showed an increase in between-network connectivity associated to ASD on the full ABIDE dataset using logistic regression. Also, observe that the connectivity for subject sex is highly correlated with the connectivity for the ASD group. Although interactions between subject sex and ASD have been identified^37^, we believe this highlights a possible limitation of R-PLS and requires further investigation in future research.

The role of the basal ganglia in schizophrenic patients has been previously observed, particularly the decrease in connectivity between the salience network and the basal ganglia^38,39^ and the decreased connectivity between the cerebellum and basal ganglia^40^. Similarly, the importance of the thalamus in schizophrenia, identified when using the AAL atlas, is well-known^41,42^. Further, the connectivity patterns involving the default mode network have been previously reported in schizophrenic patients^43–47^.

The results for eye status during scan are also well represented in the literature. The decreased within-network connectivity for the default mode network for patients with closed eyes has been previously reported by Yan et. al.^48^, and the increased between-network connectivity for the default mode network has recently been discussed by Han et. al.^49^. Further, the observed decrease in connectivity for the occipital network agrees with Agcaoglu et. al.^50^.

The use of the VIP statistic to identify significant connections in functional connectivity has not been previously studied. We have demonstrated that this statistic can identify many functional connections that have been addressed previously in the literature, but it is not without its limitations. First, with our focus on generalising partial least squares to Riemannian manifolds, the VIP statistic does not take into account the Riemannian geometry we are considering. This is mitigated by the tangent space approximation we are performing, which directly accounts for the geometry of the data, but further research could help better generalise the VIP statistic for R-PLS. Further, the VIP statistic associates the effects of a single predictor on the full multivariate response. In situations like we consider here, this makes it difficult to determine which functional connections are associated to which outcome variable. For example, the connectivity within the default mode network is deemed significant by the VIP statistic in the ABIDE dataset, but it is unclear whether this connectivity is significance for every outcome variable or a subset of them. Work has been done to generalise the VIP statistic when the outcome variable is multivariate^51^, but further research is needed to investigate this generalisation.

The R-PLS method has shown to be generalisable over different atlases and datasets, but with a few notable differences. When analysing the COBRE dataset, we observe similar results between functional connectivity and age and subject group, but due to the different granularity of the MSDL and AAL atlases (39 ROI for MSDL and 116 for AAL) we are able to find further relationships. For example, the relationship between the within-network connectivity of the basal ganglia and age, or the role of the thalamus in schizophrenia. Comparing the AAL atlas across the COBRE and ABIDE datasets, we find that R-PLS is still the preferred model over Euclidean methods. Looking at the relationship between functional connectivity and age across both datasets, we see some similarities in the results with the differences explained by the different ages considered in each cohort^52^ (mean age for COBRE = 37 years old, mean age for ABIDE = 15.2 years old, Tables S1 and S2).

However, further work is needed to verify R-PLS in a clinical context. First, we have not been able to investigate the test-retest reliability of this method due to the cross-sectional nature of the COBRE and ABIDE studies. A thorough study of the test-retest reliability of R-PLS would be invaluable to the method and would increase its versatility for clinical studies. Second, we have not investigated the effects of different preprocessing choices for the fMRI data. It would be beneficial to the generalisability of the results from R-PLS to determine how robust they are across preprocessing choices. The different preprocessing pipelines for the ABIDE study offer one avenue to investigate this, and is a clear area of future research.

These results suggest that R-PLS can provide insight into the functional connectome and how it relates to subject phenotype data. Further, due to the specification and generality of the R-PLS model, this method is readily applicable to other imaging modalities, and in particular to multimodal imaging studies. The application of R-PLS to multimodal imaging studies is an area of future research that may help to us to understand the functional networks that make up the human connectome.

## Methods

### Data

The International Neuroimaging Data-Sharing Initiative (INDI) is an initiative set to encourage free open access to neuroimaging datasets from around the world. We consider two datasets that are accessible as a part of the INDI.

#### COBRE

The Center for Biomedical Research Excellence (COBRE) have contributed structural and functional MRI images to the INDI that compare schizophrenic patients with healthy controls^19^. The data were collected with single-shot full *k*-space echo-planar imaging with a TR of 2000 ms, matrix size of $64\times 64$ and 32 slices (giving a voxel size of $3\times 3\times 4$ mm$^3$). These data were downloaded using the python package nilearn v 0.6.2, and contains 146 subjects (Control $= 74$), each with phenotype information on subject group and age; further information is available in Table S1 of the supplementary material.

The fMRI data were preprocessed using NIAK 0.17 under CentOS version 6.3 with Octave version 4.0.2 and the Minc toolkit version 0.3.18^53^. The data were subjected to band pass filtering and nuisance regression where we removed six motion parameters, the frame-wise displacement, five slow-drift parameters, average parameters for white matter, lateral ventricles, and global signal, as well as 5 estimates for component based noise correction^54^.

For the COBRE dataset, we consider each fMRI in the MSDL atlas and the AAL atlas^22^. The MSDL atlas is a functional ROI decomposition of 39 nodes across 17 resting state networks^26^. The AAL atlas is an anatomical atlas of 116 nodes across the brain. Time series for each atlas were extracted for each ROI by taking the mean time series across the voxels in each region.

#### ABIDE

The Autism Brain Imaging Data Exchange (ABIDE) is part of the Preprocessed Connectomes Project in INDI^21^. The ABIDE data is a collection of preprocessed fMRI images from 16 international imaging sites with 539 individuals diagnosed with ASD and 573 neurotypical controls (NTC). The ABIDE initiative provides data preprocessed under four separate standard pipelines, as well as options for band-pass filtering and global signal regression.

Here we consider the 172 subjects (NTC = 98) of the New York University imaging site. We restrict to this site to reduce inter-site variation in imaging and because it is the largest individual imaging site. The data were collected with a 3 Tesla Allegra MRI using echo-planar imaging with a TR of 2000 ms, matrix size of $64\times 64$ and 33 slices (giving a voxel size of $3\times 3\times 4$ mm $^3$). The fMRI data were downloaded using the python package nilearn v 0.6.2 preprocessed using the NIAK 0.7.1 pipeline^53^. The data were subjected to: motion realignment; non-uniformity correction using the median volume; motion scrubbing; nuisance regression which removed the first principal component of 6 motion parameters, their squares, mean white matter and cerebrospinal fluid signals, and low frequency drifts measured by a discrete cosine basis with a 0.01 Hz high-pass cut-off; band-pass filtering and; global signal regression. We consider the subjects preprocessed fMRI as well as subject group, age, sex, and eye status during scan (open or closed); further information is available in Table S2 of the supplementary material.

For the ABIDE dataset, we consider each fMRI in the AAL atlas^22^, with time series were extracted by taking the mean time series across the voxels in each ROI.

### Partial least squares in Euclidean space

PLS is a predictive modelling technique that predicts a response matrix ${Y}_{n \times q}$ from a set of predictors ${X}_{n \times p}$. Originally introduced in the chemometrics literature by Wold^6^, PLS has found application in bioinformatics^55^, social sciences^56^, and neuroimaging^8,57,58^; see Rosipal and Krämer^59^ and citations therein for further examples. As an extension of multivariate multiple regression, PLS has been shown to have better predictive accuracy than multivariate multiple regression when the standard regression assumptions are met^60^. A further advantage of PLS is that it is effective when $q > n$ or $p > n$ since it performs prediction from lower dimensional latent variables, that is, PLS constructs a new set of predictor variables from *X* to predict *Y*^60^.

Let ${X}_{n \times p}$ and ${Y}_{n \times q}$ be predictor and response matrices respectively. Suppose *X* and *Y* are column centred, that is, suppose the means of each column of *X* and *Y* are 0. PLS proposes the existence of $L \le \min \{p, n\}$ latent variables such that *X* and *Y* decompose into a set of *scores matrices* ${T}_{n\times L}$ and ${U}_{n \times L}$, and *loadings matrices* ${P}_{p\times L}$ and ${Q}_{q\times L}$ with

1 $$\begin{aligned} {X}&= {T} {P}^T + {E}, \end{aligned}$$

2 $$\begin{aligned} {Y}&= {U} {Q}^T + {F}, \end{aligned}$$

where ${E}_{n\times p}$ and ${F}_{n \times q}$ are error matrices, assumed to be a small as possible^61^, and the superscript *T* denotes the matrix transpose. Further, PLS assumes that there is a diagonal matrix ${B}_{L \times L}$ with

3 $$\begin{aligned} {U} = {T} {B} + {H}_{n\times L}, \end{aligned}$$

where *H* is a matrix of residuals. Equations (1) and (2) are called the *outer relationships* while Eq. (3) defines the *inner relationship* that connects *X* and *Y*. Combining the inner relationship and the outer relationship for *Y* gives

$$\begin{aligned} {Y} = {T} {B} {Q}^T + ({H}{Q}^T + {F}), \end{aligned}$$

which highlights that *Y* is a regression on the latent scores *T*. Further, notice that the error in *Y* is given by ${H}{Q}^T + {F}$, that is, error in *Y* is a combination of error inherent to the response data (*F*) and error from the estimation of the inner relationship (${H}{Q}^T$). The inclusion of the residual matrix *H* can complicate discussion of the PLS method, so it is common to consider the estimated inner relationship ${{\hat{U}}} \approx {T} {B}$ instead^61,62^.

Estimation of the PLS model (Eqs. 1–3) is commonly done through the non-linear iterative partial least squares (NIPALS) algorithm (Algorithm S1 in the supplementary material). The inputs for the NIPALS algorithm are the data matrices *X* and *Y* and the pre-specified number of latent variables *K*; noting that the true number of latent variables *L* is unknown, the value *K* can be chosen with methods such as cross validation. The NIPALS algorithm outputs estimates of the scores, loadings, and regression coefficients as well as matrices ${W}_{p\times K}$ and ${C}_{q\times K}$ known as the weights. The weight matrices *W* and *C* are linear transformations of *P* and *Q* that more efficiently fit the PLS model and are defined within the NIPALS algorithm; see the supplementary material S1 for further information. Using the results of the NIPALS algorithm and Eqs. (1)–(3), we can write

$$\begin{aligned} {{\hat{Y}}} = {X}{{\hat{\beta }}}_{PLS} \end{aligned}$$

where

4 $$\begin{aligned} {{\hat{\beta }}}_{PLS} = {W}({P}^T{W})^{-1}{B}{C}^T \end{aligned}$$

is the matrix of regression coefficients. Using ${{\hat{\beta }}}_{PLS}$ we see that PLS is a linear regression technique similar to ordinary least squares and ridge regression.

#### Cross validation

We choose the optimal number of latent variables *K* for each PLS model through ten-fold cross validation^24^. To do this, we split each dataset into ten equal subsets $C_1, C_2 \dots , C_{10}$ stratified by subject group (schizophrenia or ASD). For each subset $C_i$, $i=1, 2, \dots , 10$, we train the PLS models on the remaining nine subsets for each value of ${\hat{K}} = 1, 2, \dots , 50$, using the phenotype data as the response variables and the functional connectivity as the predictors. We then predict on the subset $C_i$ to calculate the test RMSE. By taking the average RMSE over all cross validation folds, we get an estimate of the test RMSE for the model. If $K^*$ is the value of ${\hat{K}}$ that returns the minimum cross validated RMSE, the optimal *K* for our model is ${K}\le K^*$ such that the cross validated RMSE for *K* is within one standard error of the cross validated RMSE for $K^*$.

#### The VIP statistic

To determine significant predictors of the response variables in the PLS model, we use the VIP statistic^18^. Suppose there are *p* predictor variables, *q* response variables, and *K* latent variables extracted using NIPALS. Following Tennenhaus^63^, the VIP statistic for the *j*th predictor variable is

5 $$\begin{aligned} \textrm{VIP}_j = \sqrt{\frac{ p }{ \textrm{Rd}({Y}, {T}) }\sum \limits _{k = 1}^K \textrm{Rd}({Y}, {t_k}) \left( w_{jk} \right) ^2}\,, \end{aligned}$$

where ${t}_k$ is the $k^{th}$ column of the score matrix *T*, $w_{jk}$ is the *k*th weight for the *j*th predictor, $\textrm{Rd}({Y}, {t_k}) = \frac{1}{q} \sum \nolimits _{i = 1}^q \textrm{cor}({Y}_i, {t}_k)^2$, and $\textrm{Rd}({Y}, {T}) = \sum \nolimits _{k = 1}^K \textrm{Rd}({Y}, {t_k})$. The coefficient $\textrm{cor}({Y}_i, {t}_k)^2$ is the squared correlation between the *j*th response variable and the *k*th score. The denominator $\textrm{Rd}({Y}, {T})$ in Eq. (5) measures the proportion of variance in *Y* explained by *T*, and the numerator $\textrm{Rd}({Y}, {t_k}) (w_{jk})^2$ measures the proportion of variance in *Y* described by the *k*th latent variable that is explained by the *j*th predictor^64^. Thus the VIP statistic measures the influence of each predictor on the explained variation in the model^65^.

Commonly, the “greater than one”  rule is used to find predictors significantly associated with the response. However, this rule is motivated by the mathematical properties of $\textrm{VIP}_j$ rather than statistical properties^64^. Thus, we use a permutation test to determine significance of $\textrm{VIP}_j$. This is an alternative to Afanador et. al.^66^ who used $95\%$ jackknife confidence intervals to determine significance of VIP .

Specifically, for each predictor variable *j* we permute the values *H* times. For each permutation $h =1, 2, \dots , H$ we refit the PLS model and calculate $\textrm{VIP}_{j, h}$. The *P*-value for the *j*th VIP score is then

6 $$\begin{aligned} \text {{P-}value}_{j} = \frac{\#\, \left\{ \textrm{VIP}_{j, h} > \textrm{VIP}_{j} \right\} }{H}\,. \end{aligned}$$

For our data, the predictors are functional connectivity matrices. Thus, we know a priori that the diagonal elements are uninformative since they are identically one. Hence, if predictor *j* describes a diagonal element we set ${{P}{\text {-value}}}_{j} = 1$ for all *i*. To account for the multiple comparisons problem, we adjust all *P*-values using the false discovery rate^67^ and determine significance at a significance level of $\alpha = 0.05$.

### Mathematical preliminaries

#### Riemannian manifolds

Intuitively speaking, a Riemannian manifold *M* is a space where we can perform calculus, measure distances, and measure angles between tangent vectors. More specifically, a smooth *d*-dimensional manifold *M* is a connected, Hausdorff, second countable topological space that is covered by a set of coordinate charts $\{(U_i, \varphi _i:U_i\rightarrow {\mathbb {R}}^d)\}_{i \in I}$, defined by some indexing set *I*, such that every point in *M* belongs to a $U_i$ for some $i\in I$ and the intersection maps $\varphi _i \circ \varphi _j^{-1}$ are smooth as maps ${\mathbb {R}}^d \rightarrow {\mathbb {R}}^d$ for every $i, j \in I$. These coordinate charts make the space *M* “locally Euclidean” in the sense that every point has a neighbourhood that looks like Euclidean space. Since concepts from differential calculus are local in nature, the construction of a smooth manifold allows us to perform calculus on these more general spaces.

An important concept in the study of manifolds is the tangent bundle $TM = \bigsqcup _{a \in M} T_{a}M$, where $T_{a}M$ is the tangent space at *a*. The space $T_{a}M$ is defined as the set of equivalence classes of curves through *a* such that $\gamma _1$ and $\gamma _2$ are equivalent if $\gamma _1'(0) = \gamma _2'(0)$, where the prime denotes the derivative. Then $T_{a}M$ is a vector space that generalises the notion of vectors tangent to a surface to arbitrary smooth manifolds.

A *Riemannian* manifold is a manifold *M* together with a smooth map $g:M\times TM\times TM \rightarrow {\mathbb {R}}$ such that $g(a,\cdot , \cdot ) = g_a:T_{a}M \times T_{a}M\rightarrow {\mathbb {R}}$ is an inner product for every $a\in M$. The Riemannian metric *g* allows us to measure angles between tangent vectors and measure distances between points on the manifold *M*. Further, *g* is used to define geodesics (locally length minimising curves) $\gamma :[t_0, t_1]\rightarrow M$ between two points $a, b \in M$. We only consider complete Riemannian manifolds here, which are spaces where every geodesic $\gamma$ has domain ${\mathbb {R}}$.

Through geodesics we get the concepts of the Riemannian exponential and logarithm maps which allow us to smoothly move between the manifold and the tangent space. The Riemannian exponential at a point $a\in M$ is a map ${{\,\textrm{Exp}\,}}_{a}:T_{a}M\rightarrow M$ defined by ${{\,\textrm{Exp}\,}}(a, \cdot )(\gamma ) = {{\,\textrm{Exp}\,}}_{a}(\gamma ) = \gamma (1)$, where $\gamma$ is a geodesic such that $\gamma (0) = a$. The Riemannian exponential is a smooth map that is locally diffeomorphic and hence has a local inverse denoted ${{\,\textrm{Log}\,}}(a, \cdot ) = {{\,\textrm{Log}\,}}_{a} : M\rightarrow T_{a}M$ defined by ${{\,\textrm{Log}\,}}_{a}(b) = \gamma '(0)$ where $\gamma (t)$ is a geodesic from *a* to *b*. For a point $b \in M$ close to *a*, we think of ${{\,\textrm{Log}\,}}_{a}(b)$ as the shortest initial velocity vector based at *a* pointing in the direction of *b*. Further information on Riemannian manifolds can be found in the books by Lee^68–70^ or do Carmo (1992)^71^. An accessible introduction for medical imaging can be found in the book edited by Pennec et. al.^10^.

#### Fréchet mean

To capture the centre of data on a manifold we consider the Fréchet (or intrinsic) mean of data $X_1, X_2, \dots , X_n \in M$. First, consider the Riemannian distance between two close points $X_1, X_2\in M$ defined by

$$\begin{aligned} d_g(X_1, X_2) = \left\| {{\,\textrm{Log}\,}}_{X_1}(X_2)\right\| , \end{aligned}$$

where $\Vert \cdot \Vert$ is the norm in $T_{X_1}M$ induced by the Riemannian metric. By generalising the sum of squared distances definition of the arithmetic mean, the Fréchet mean^72^ is given by

$$\begin{aligned} \mu _X = {{\,\textrm{argmin}\,}}\sum _{i=1}^n d_g(X_i, \mu _X)^2\,. \end{aligned}$$

We solve for $\mu _X$ using gradient decent^10^; see Algorithm S2 in the supplementary material for further information.

#### The affine invariant geometry for symmetric positive definite matrices

Let $GL_R{\mathbb {R}}$ be the set of $R\times R$ real invertible matrices. The set of symmetric positive definite matrices is defined by

$$\begin{aligned} S^+_R = \left\{ {A}\in GL_R{\mathbb {R}}: {A}^T = {A} \text { and } {v}^T {A} {v} > 0 \text { for all } {v} \in {\mathbb {R}}^{R} \backslash \{{0}\} \right\} \,, \end{aligned}$$

where superscript *T* denotes matrix transpose. The set $S_R^+$ is a smooth manifold, which can be easily seen by embedding $S_R^+$ into ${\mathbb {R}}^{R(R+1)/2}$ as a convex cone. This construction shows that the tangent space at each ${A} \in S_R^+$ is given by the set of symmetric $R\times R$ matrices.

However, $S_R^+$ has an interesting intrinsic geometry known as the affine-invariant geometry^9^. Under the affine invariant geometry $S_R^+$ becomes a complete Hadamard manifold—a Riemannian manifold of non-positive curvature where ${{\,\textrm{Exp}\,}}_{{A}}$ is a diffeomorphism for every ${A} \in S_{R}^+$.

The affine-invariant metric *g* is defined by

$$\begin{aligned} g_{{A}}({U}, {V}) = {{\,\textrm{Tr}\,}}\left( {U} {A}^{-1} {V} {A}^{-1} \right) \,, \end{aligned}$$

where ${A} \in S_R^+$, ${U}, {V} \in T_{{A}}S_R^+$, and ${{\,\textrm{Tr}\,}}$ denotes the trace operator. Using *g*, we can calculate the Riemannian distance between ${A}, {B} \in S_R^+$ as

$$\begin{aligned} d_g({A}, {B})^2 = \sum _{r = 1}^R \left( \log \left( \sigma _r\left( {A}^{-1/2} {B} {A}^{-1/2} \right) \right) \right) ^2\,, \end{aligned}$$

where $\sigma _r\left( {A}^{-1/2} {B} {A}^{-1/2} \right)$ are the eigenvalues of ${A}^{-1/2} {B} {A}^{-1/2}$, $r= 1, 2, \dots , R$. Further, letting ${A}, {B} \in S_R^+$ and ${U} \in T_{{A}}S_R^+$, we get

$$\begin{aligned} {{\,\textrm{Exp}\,}}_{{A}}({U}) = {A}^{1/2} {{\,\textrm{Exp}\,}}\left( {A}^{-1/2}{U}{A}^{-1/2} \right) {A}^{1/2} \end{aligned}$$

and

$$\begin{aligned} {{\,\textrm{Log}\,}}_{{A}}({B}) = {A}^{1/2} {{\,\textrm{Log}\,}}\left( {A}^{-1/2}{B}{A}^{-1/2} \right) {A}^{1/2}\,, \end{aligned}$$

where ${{\,\textrm{Exp}\,}}$ and ${{\,\textrm{Log}\,}}$ are the matrix exponential and logarithm respectively. The Riemannian distance, exponential, and logarithm are essential in the definition and fitting of the R-PLS model defined below.

### Riemannian PLS

Let *M* and *N* be complete Riemannian manifolds. Let $X_1, X_2, \dots , X_n \in M$ and $Y_1, Y_2, \dots , Y_n \in N$, and let $\mu _X$ and $\mu _Y$ denote the respective Fréchet means. Let $L \le \min \{ \dim (M), n \}$. The R-PLS model proposes the existence of loadings ${p}_1, {p}_2, \dots , {p}_L \in T_{\mu _X}M$ and ${q}_1, {q}_2, \dots , {q}_L \in T_{\mu _Y}N$ such that, for each subject $i = 1, 2, \dots , n$, there are scores $t_{i1}, t_{i2}, \dots , t_{iL} \in {\mathbb {R}}$ and $u_{i1}, u_{i2}, \dots , u_{iL} \in {\mathbb {R}}$ with

7 $$\begin{aligned} X_i&={{\,\textrm{Exp}\,}}\left( {{\,\textrm{Exp}\,}}_{\mu _X}\left( \sum _{l = 1}^L t_{il} {p}_l\right) , {e}_i\right) \, , \end{aligned}$$

8 $$\begin{aligned} Y_i&= {{\,\textrm{Exp}\,}}\left( {{\,\textrm{Exp}\,}}_{\mu _Y}\left( \sum _{l = 1}^L u_{il} {q}_l \right) , {f}_i\right) \, , \text { and }\end{aligned}$$

9 $$\begin{aligned} {\hat{u}}_{il}&= {\hat{\beta }}_{0l} + {\hat{\beta }}_{1l} t_{il} \text { for all }l = 1, 2, \dots , L\text { and }i = 1, 2, \dots , n\,, \end{aligned}$$

where ${e}_i \in T_{{{\,\textrm{Exp}\,}}_{\mu _X}\left( \sum _{l = 1}^L t_{il} {p}_l\right) } M$ and ${f}_i \in T_{{{\,\textrm{Exp}\,}}_{\mu _Y}\left( \sum _{l = 1}^L u_{il} {q}_l \right) }M$ are error vectors with $\Vert {e}_i\Vert$, $\Vert {f}_i\Vert$ small. Equations (7) and (8) are the *outer relationships* for Riemannian data, and Eq. (9) is the *inner relationship* connecting our response and predictor. Note that, since the Riemannian exponential map on Euclidean space is vector addition, if $M = {\mathbb {R}}^p$ and $N = {\mathbb {R}}^q$ the R-PLS model (Eqs. 7–9) reduce to the standard PLS model (Eqs. 1–3).

One approach to fitting R-PLS is by directly generalising NIPALS (Algorithm S1) to Riemannian manifolds, but this becomes computationally intensive and fails to converge for sample sizes above 20 (see Ryan^51^ for more details). Instead, we propose a tangent space approximation to fitting R-PLS when our data is close to the Fréchet mean, similar to methods such as Riemannian canonical correlations analysis^13^ and principal geodesic analysis^11^.

The tNIPALS algorithm (Algorithm 1) works by first linearising the manifold data in a neighbourhood of the Fréchet mean using the Riemannian logarithm (see supplementary material S1 for further information), and then applying the Euclidean NIPALS algorithm to the linearised data which is now vector-valued. Thus, tNIPALS provides a combination of the simplicity and efficiency of Euclidean NIPALS with the geometry of the Riemannian manifold.

**Figure:**
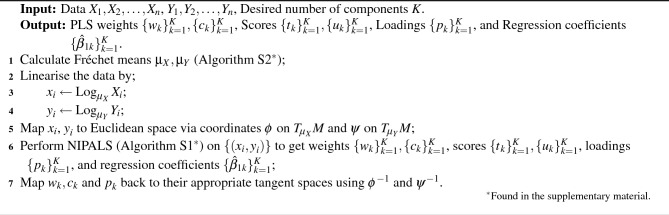
Algorithm 1: Tangent non-linear iterative partial least squares.

The tNIPALS algorithm provides a more general approach to Wong et. al.’s^14^ method for constructing predictors from functional connectivity matrices to predict ASD using PLS and logistic regression by considering a Euclidean response and symmetric positive definite predictor. Similarly, Zhang and Liu^16^ and Chu et al.^15^ also proposed PLS methods using the affine-invariant geometry for symmetric positive definite matrices that is generalised by tNIPALS. Further, the tNIPALS algorithm for R-PLS is closely related to the PLS method for symmetric positive definite matrices offered by Perez and Gonzalez-Farias^17^, where they also propose linearising symmetric positive definite matrices in the affine-invariant geometry to fit the PLS model.

### Supplementary Information

Supplementary Information.

## Data Availability

The data and r package (spdMatrices) used to complete this work are available on GitHub (Matthew-Ryan1995/Riemannian-statistical-techniques-with-applications-in-fMRI). The code to perform the analyses and generate the figures is also found on GitHub (Matthew-Ryan1995/R-PLS-for-functional-connectivity).
