# Supplementary material for: Capturing functional connectomics using Riemannian partial least squares
Source: Sci Rep. 2023 Oct 13;13:17386. doi: 10.1038/s41598-023-44687-2 (PMC10576060; doi:10.1038/s41598-023-44687-2)
Supplement: Supplementary file 1 — Supplementary Information. [file 41598_2023_44687_MOESM1_ESM.pdf]

Supplementary material to: Capturing functional connectomics using Riemannian partial least squares

Matthew Ryan[1,*], Gary Glonek[1], Jono Tuke[1], and Melissa Humphries[1]

[1]The University of Adelaide, School of Computer and Mathematical Sciences, Adelaide, 5005, Australia
[*]matthew.ryan@adelaide.edu.au

Summary statistics

For the COBRE dataset, we have phenotype information on subject group, sex, handedness, and age (Table S1). In our analysis, we only consider subject group and age as these were previously found to be associated with the distribution of the functional connectivity matrices in this dataset[1].

For the ABIDE dataset, we have phenotype information on subject group, sex, eye status during scan, age, and three measures of intellegence quotient (full scale, visual, and performance; Table S2). In our analysis, we only consider subject group, sex, eye status and age as these were previously found to be associated with the distribution of the functional connectivity matrices in this dataset[1].

Table S1. Summary statistics for the COBRE dataset. Numeric values are presented as mean (sd), and count values are presented as *N* (%).

	Patient	Control	Total
Count	72 (49.3)	74 (50.7)	146 (100)
Female	14 (19.4)	23 (31.1)	37 (25.3)
Left-handed	10 (13.9)	1 (1.4)	11 (7.5)
Age	38.2 (13.9)	35.8 (11.6)	37 (12.8)

Table S2. Summary statistics for the ABIDE dataset. Numeric values are presented as mean (sd), and count values are presented as *N* (%).

	Autism Spectrum Disorder	Neurotypical Controls	Total
Count	75 (43)	98 (57)	172 (100)
Female	10 (13.5)	26 (26.5)	36 (20.9)
Closed eyes	10 (13.5)	15 (15.3)	25 (14.5)
Age	14.8 (7.1)	15.8 (6.2)	15.3 (6.6)
Full scale IQ	107.4 (16.4)	113.4 (13.1)	110.8 (14.9)
Visual IQ	105.1 (15.9)	113.2 (12.6)	109.7 (14.6)
Performance IQ	108.6 (17.3)	110.4 (13.6)	109.6 (15.2)

NIPALS

Let $X_{n \times p}$ and $Y_{n \times q}$ be column centred predictor and response matrices respectively. Consider the partial least squares (PLS) model

$$
\begin{aligned}
X &= TP^T + E\,, \\
Y &= UQ^T + F \\
U &= TB + H_{n \times L}\,.
\end{aligned}
$$

The scores T, U, loadings P, Q, and regression matrix B can be iteratively calculated using the non-linear iterative partial least squares (NIPALS) algorithm (Algorithm S1). Further, Algorithm S1 returns the weights matrices W and C which allow PLS to more efficiently predict on new data.

Algorithm S1: Non-linear iterative partial least squares (NIPALS)

Input: Predictor matrix X, Response matrix Y, number of components K

Output: Weights W, C, Scores T, U, Loadings P, Q, Regression matrix B

$X^1 \leftarrow X$;

$Y^1 \leftarrow Y$;

for k = *1, 2, . . . ,* K **do**

  Calculate the scores and weights;

  $u \leftarrow Y^k[,1]$;

  repeat

    $w \leftarrow (X^k)^T u / u^T u$;

    $w \leftarrow w / \|w\|$;

    $t \leftarrow X^k w$;

    $c \leftarrow (Y^k)^T t / t^T t$;

    $c \leftarrow c / \|c\|$;

    $u \leftarrow Y^k c$;

  until *convergence*;

  Calculate the loadings;

    X^k-loadings: $p \leftarrow (X^k)^T t / t^T t$;

    Y^k-loadings: $q \leftarrow (Y^k)^T u / u^T u$;

  The regression step;

    $b_k \leftarrow u^T t / t^T t$;

  The deflation step;

    $X^{k+1} \leftarrow X^k - tp^T$;

    $Y^{k+1} \leftarrow Y^k - b_k t c^T$;

  Save;

    $W[,k] = w$;

    $C[,k] = c$;

    $T[,k] = t$;

    $U[,k] = u$;

    $P[,k] = p$;

    $Q[,k] = q$;

    $B[k,k] = b_k$;

end

The Fréchet mean

Let M be a Riemannian manifold and $X_1, X_2, \dots, X_n \in M$ be data. Denote the Riemannian distance function by d_g and recall the Fréchet mean is given by

$$\mu_X = \operatorname{argmin} \sum_{i=1}^{n} d_g(X_i, \mu_X)^2 \,.$$

Following do Carmo (1992)[2] the gradient of d_g is given by

$$\operatorname{grad}_x d_g(y,x) = -2\operatorname{Log}(x,y)\,.$$

Gradient descent can then be applied to calculate the Fréchet mean using Algorithm S2.

Algorithm S2: Gradient descent to calculate the Fréchet mean on the Riemannian manifold M. This is Algorithm 2.1 from Pennec *et. al.* (2019)[3] with an adapted step size τ.

Input: Data $y_1, y_2, \dots, y_n \in M$, tolerance ε, step size τ
Output: $\mu_Y \in M$, the Fréchet mean

Set $\mu_Y^{(0)} = y_1$;
while $\varepsilon^{(k)} > \varepsilon$ **do**
    $v = \frac{\tau}{n} \sum_{i=1}^{n} \operatorname{Log}_{\mu_Y^{(k)}}(y_i)$;
    if $\|v\|_{\mu_Y^{(k)}} > \varepsilon^{(k)}$ **then**
        $\tau = \tau/2$;
        $\mu_Y^{(k+1)} = \mu_Y^{(k)}$
    else
        $\mu_Y^{(k+1)} = \operatorname{Exp}_{\mu_Y^{(k)}}(v)$;
        $\varepsilon^{(k+1)} = \|v\|_{\mu_Y^{(k)}}$
    end
end

Linearising functional connectivity matrices in the affine invariant geometry

To fit the Riemannian partial least squares (R-PLS) model using tangent non-linear iterative partial least squares (tNIPALS), we first need to linearise the manifold data at the Fréchet mean to get a vector representation, on which we can perform Euclidean NIPALS (Algorithm S1). We discuss this further for the symmetric positive definite matrices S_R^+ equipped with the affine invariant metric following the exposition in Pennec *et. al.* (2019)[3].

Let $X_1, X_2, \ldots, X_n \in S_R^+$ and let μ_X denote their Fréchet mean. We first define the function $\text{Vec} : T_I S_R^+ \to \mathbb{R}^{R(R+1)/2}$. Recall that an element $U \in T_I S_R^+$ is a symmetrix matrix, which we write

$$U = \begin{bmatrix} u_{11} & u_{12} & \cdots & u_{1R} \\ u_{12} & u_{22} & \cdots & u_{2R} \\ \vdots & \vdots & \ddots & \vdots \\ u_{1R} & u_{2R} & \cdots & u_{RR} \end{bmatrix}.$$

Define

$$\text{Vec}(U) = \left(u_{11}, u_{22}, \ldots, u_{RR}, \sqrt{2}u_{12}, \sqrt{2}u_{13}, \ldots, \sqrt{2}u_{RR}\right)^T.$$

Then Vec is a smooth isometry between $T_I S_R^+$ and $\mathbb{R}^{R(R+1)/2}$. Using the affine invariant geometry, Vec can be extended to μ_X by

$$\text{Vec}_{\mu_X}(U) = \text{Vec}\left(\mu_X^{-1/2} U \mu_X^{-1/2}\right),$$

where $U \in T_{\mu_X} S_R^+$ and $\mu_X^{-1/2}$ is the inverse of the symmetric square root of μ_X. Recalling that

$$\text{Log}_{\mu_X}(X_i) = \mu_X^{1/2} \,\text{Log}\left(\mu_X^{-1/2} X_i \mu_X^{-1/2}\right) \mu_X^{1/2},$$

takes X_i to $T_{\mu_X} S_R^+$, we can linearise our data by mapping

$$X_i \mapsto \text{Vec}_{\mu_X}\left(\text{Log}_{\mu_X}(X_i)\right).$$

This linearisation can be visualised with the following commutative diagram.

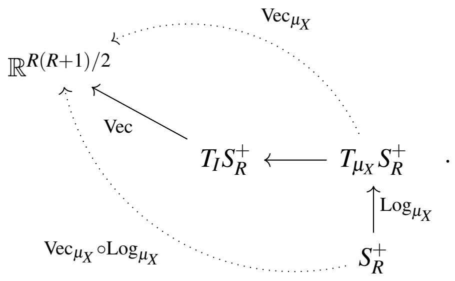

Supplementary Figures

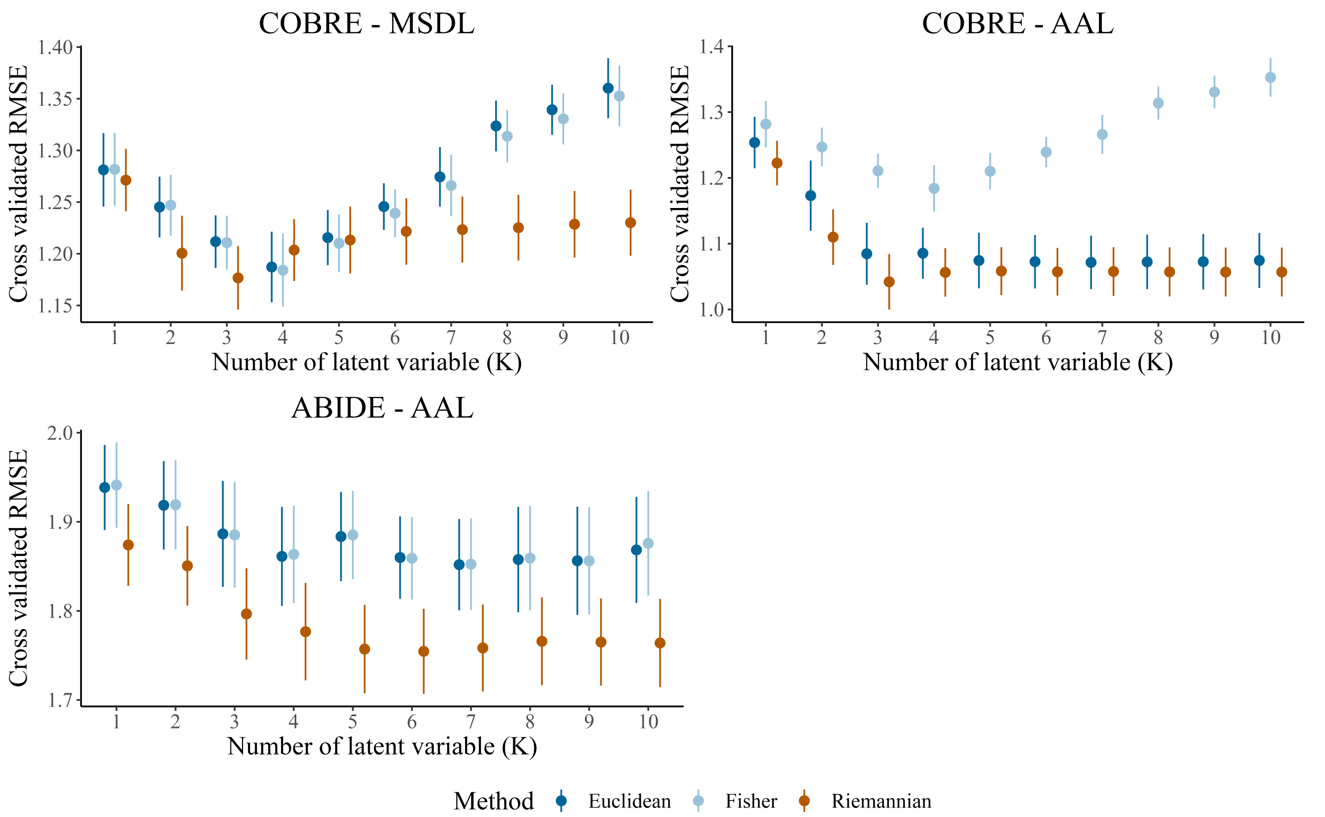

Figure S1. Ten-fold cross validation results for fitting Riemannian partial least squares (R-PLS), and PLS with Euclidean and Fisher predictors for both the COBRE and ABIDE datasets, and the multi-subject dictionary learning (MSDL) and automated anatomic labelling (AAL) atlases. The x-axis shows the number of latent variables K in the model (limited to $K \leq 10$ for visual clarity), and the y-axis shows the cross-validated root mean square error (RMSE). The points on the plots are the mean RMSE from the cross validation, and the lines represent ± 1 standard error from the mean. The dark blue is the Euclidean method, the light blue is the Fisher method, and the orange is the Riemannian method. In each case, we see that R-PLS outperforms the other methods.

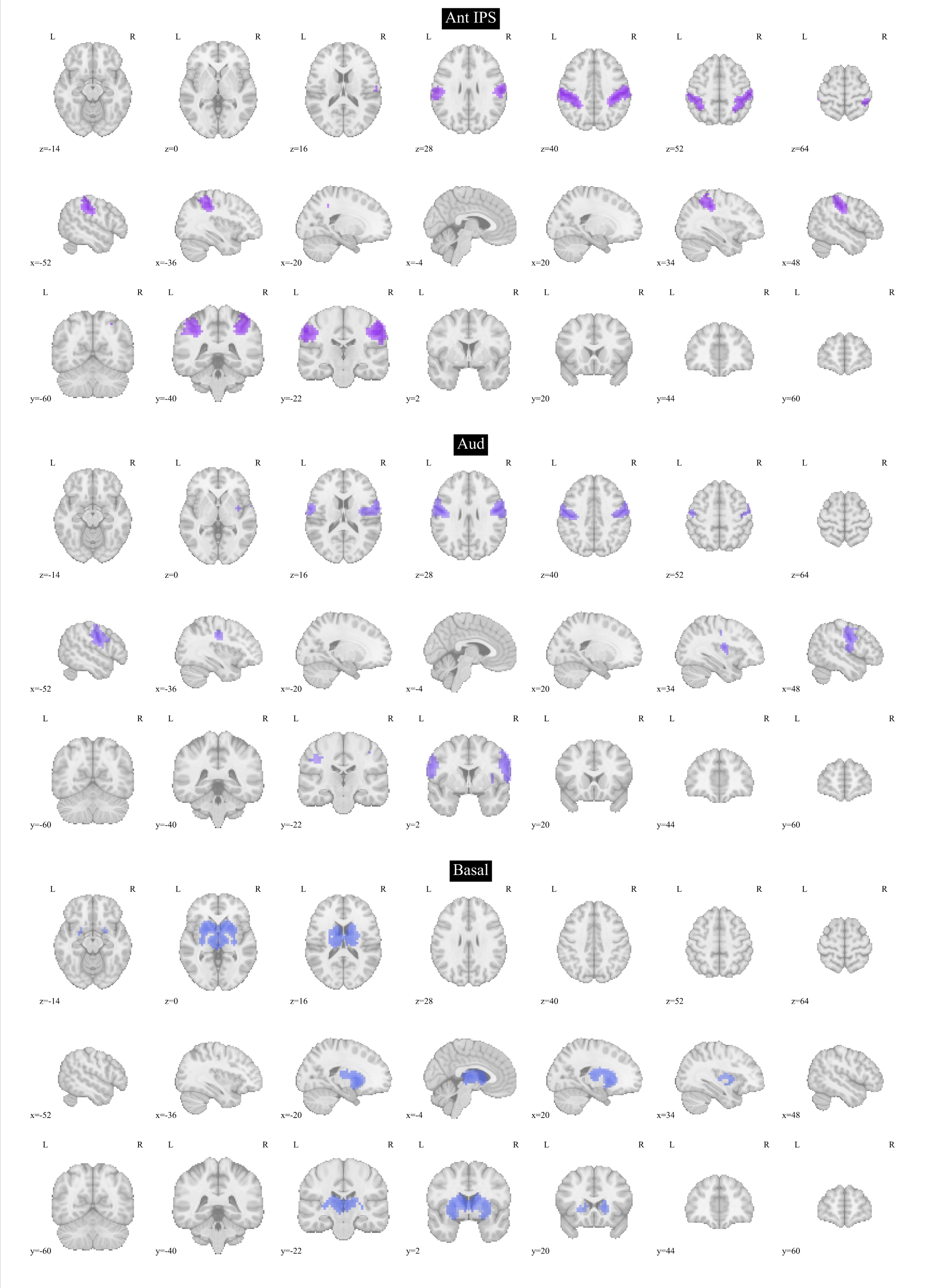

Figure S2. Resting state networks in the multi-subject dictionary learning (MSDL) atlas. Top to bottom we see the Ant IPS = Anterior Intraparietal Sulcus, Aud = Auditory, and Basal = Basal Ganglia networks.

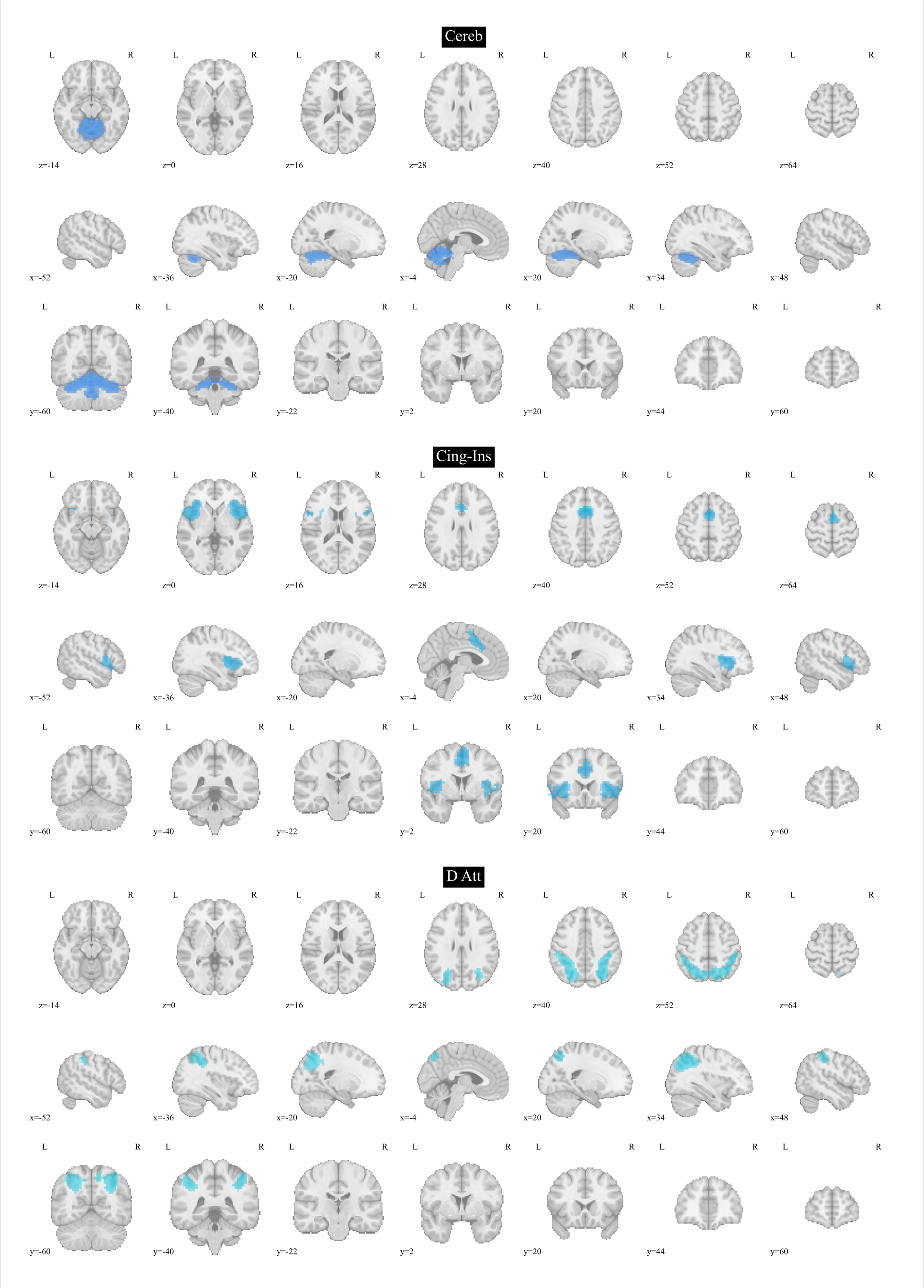

Figure S3. Resting state networks in the multi-subject dictionary learning (MSDL) atlas. Top to bottom we see the Cereb = Cerebellum, Cing-Ins = Cingulate-Insula, and D Att = Dorsal Attention Networks.

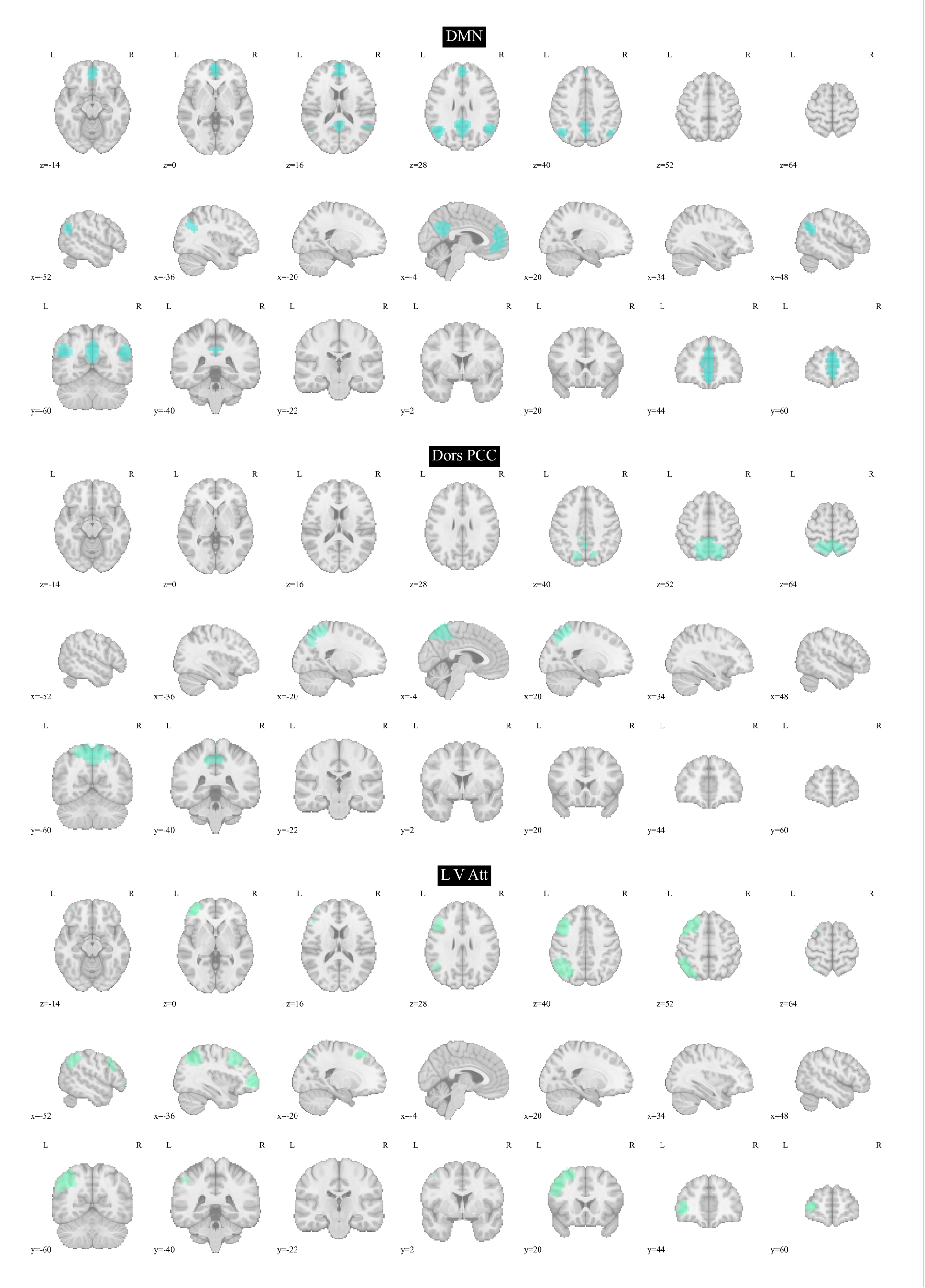

Figure S4. Resting state networks in the multi-subject dictionary learning (MSDL) atlas. Top to bottom we see the DMN = Default Model Network, Dors PCC = Dorsal Posterior Cingulate Cortex, and L V Att = Left Ventral Attention Networks.

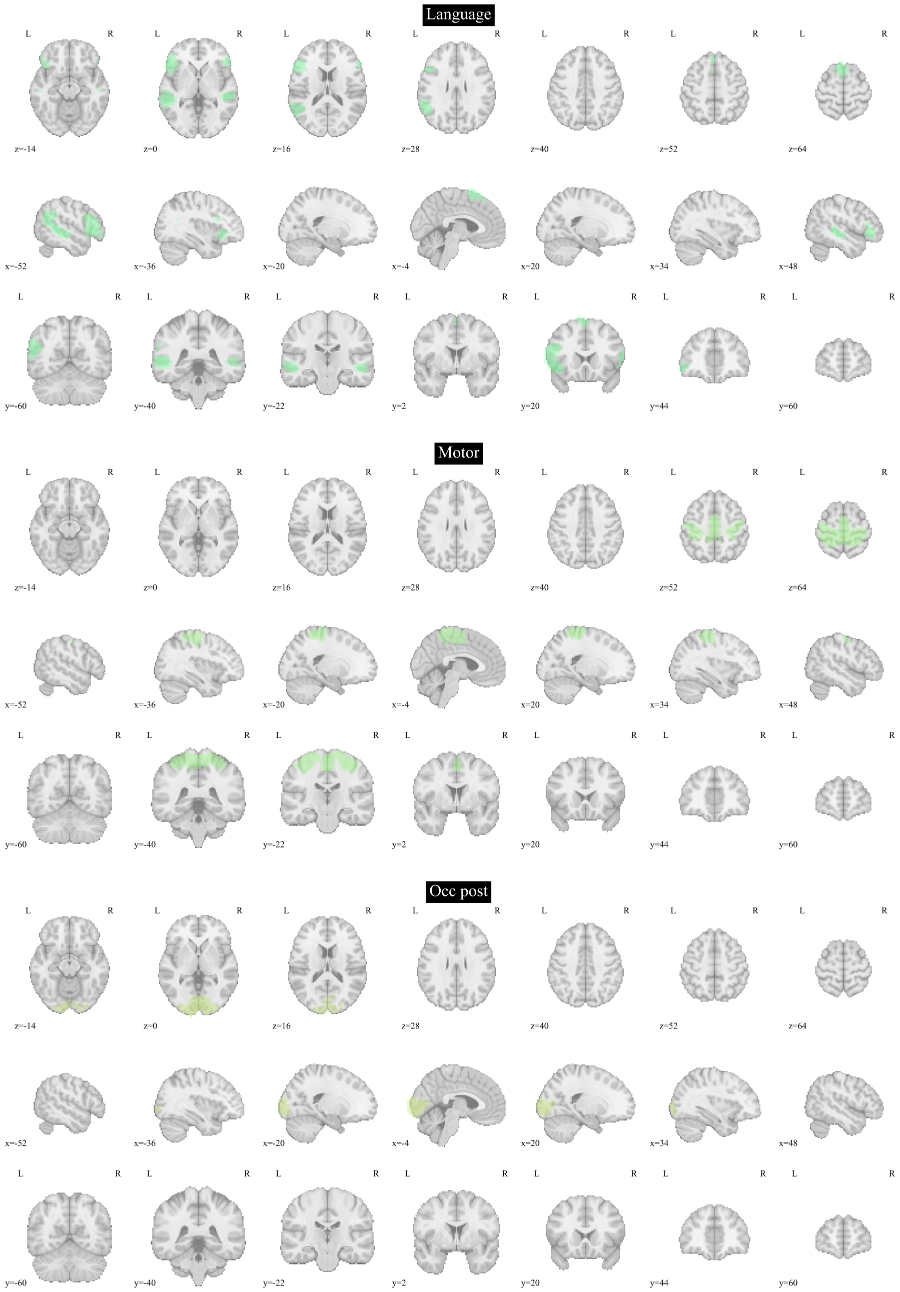

Figure S5. Resting state networks in the multi-subject dictionary learning (MSDL) atlas. Top to bottom we see the Language = Language, Motor = Motor , and Occ Post = Occipital Posterior networks.

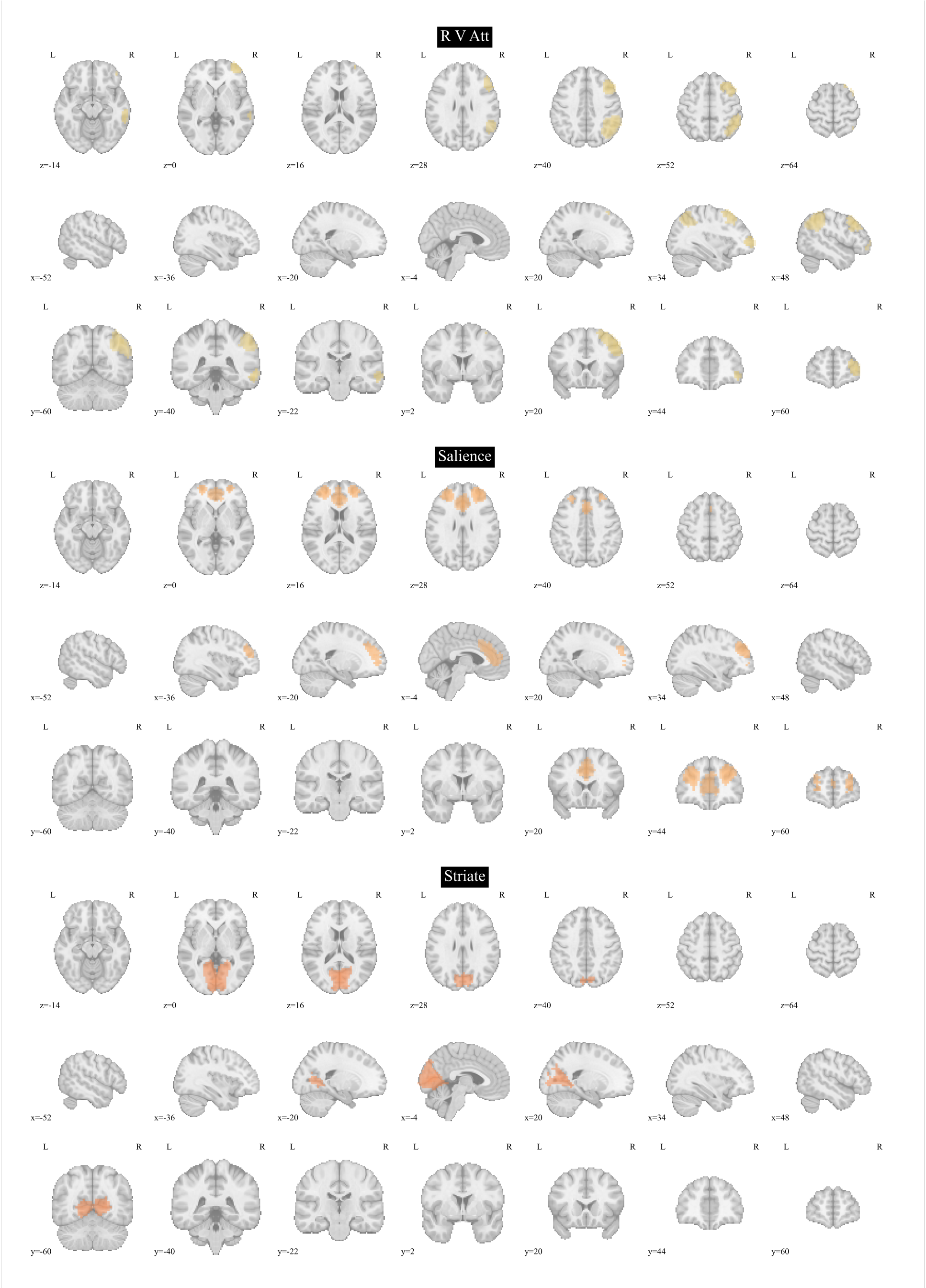

Figure S6. Resting state networks in the multi-subject dictionary learning (MSDL) atlas. Top to bottom we see the R V Att = Right Ventral Attention Network, Salience = Salience Network, and Basal = Basal Ganglia networks.

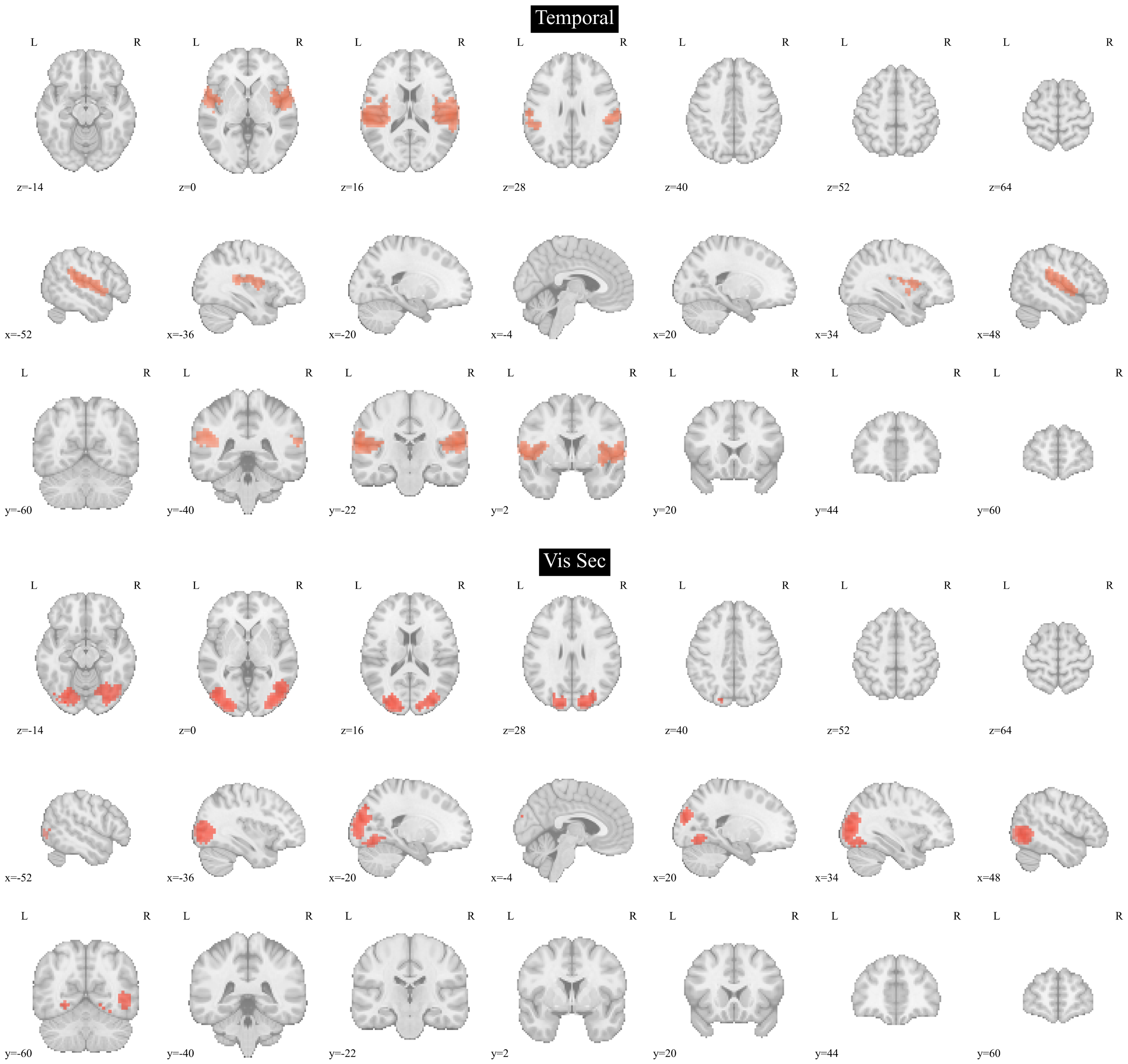

Figure S7. Resting state networks in the multi-subject dictionary learning (MSDL) atlas. Top to bottom we see the Temporal = Temporal Network and Vis Sec = Secondary Visual Cortex Network.

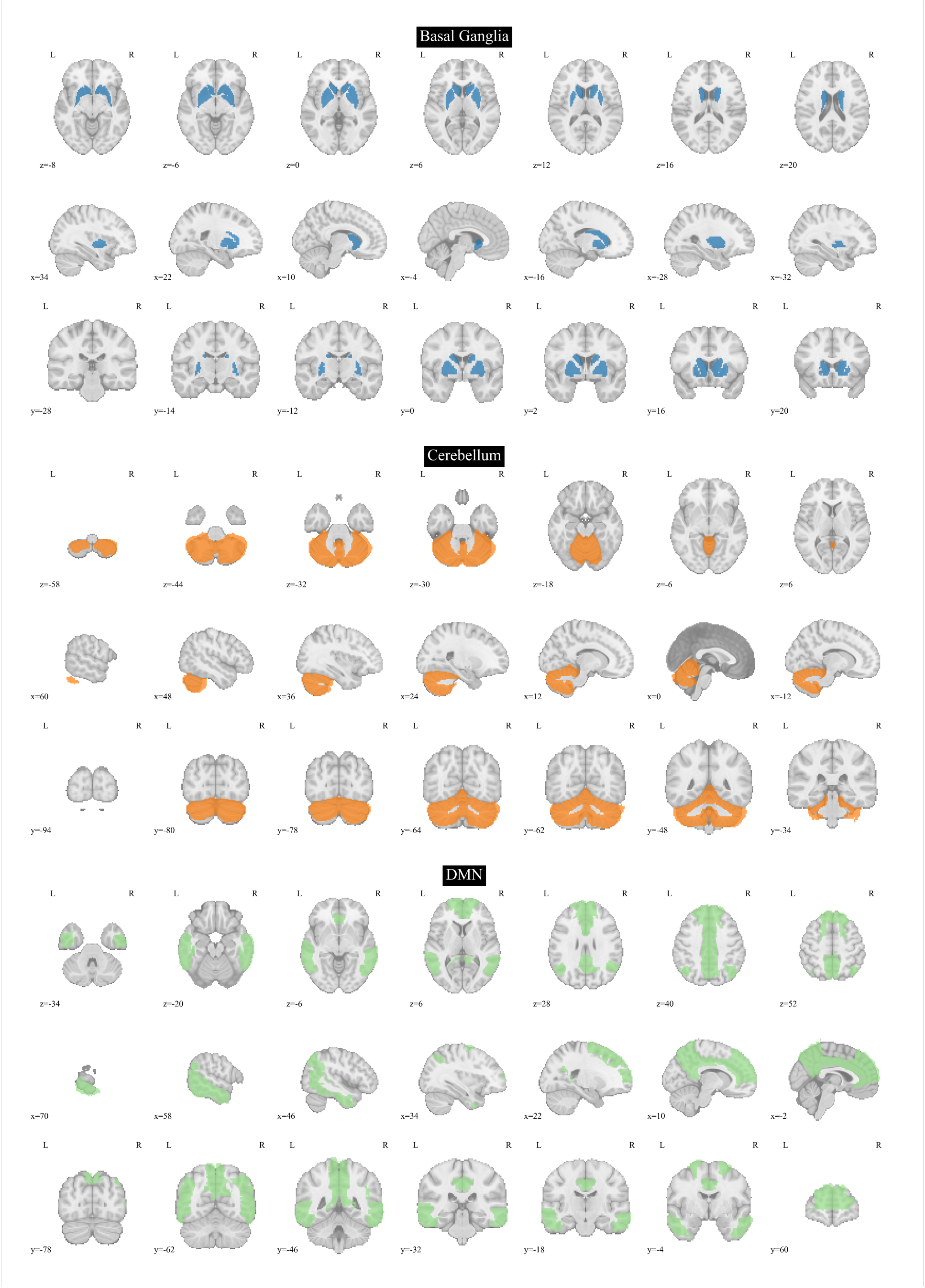

Figure S8. Resting state networks in the automated anatomic labelling (AAL) atlas. Top to bottom we see the Basal Ganglia, Cerebellum, and DMN=Default Mode Network.

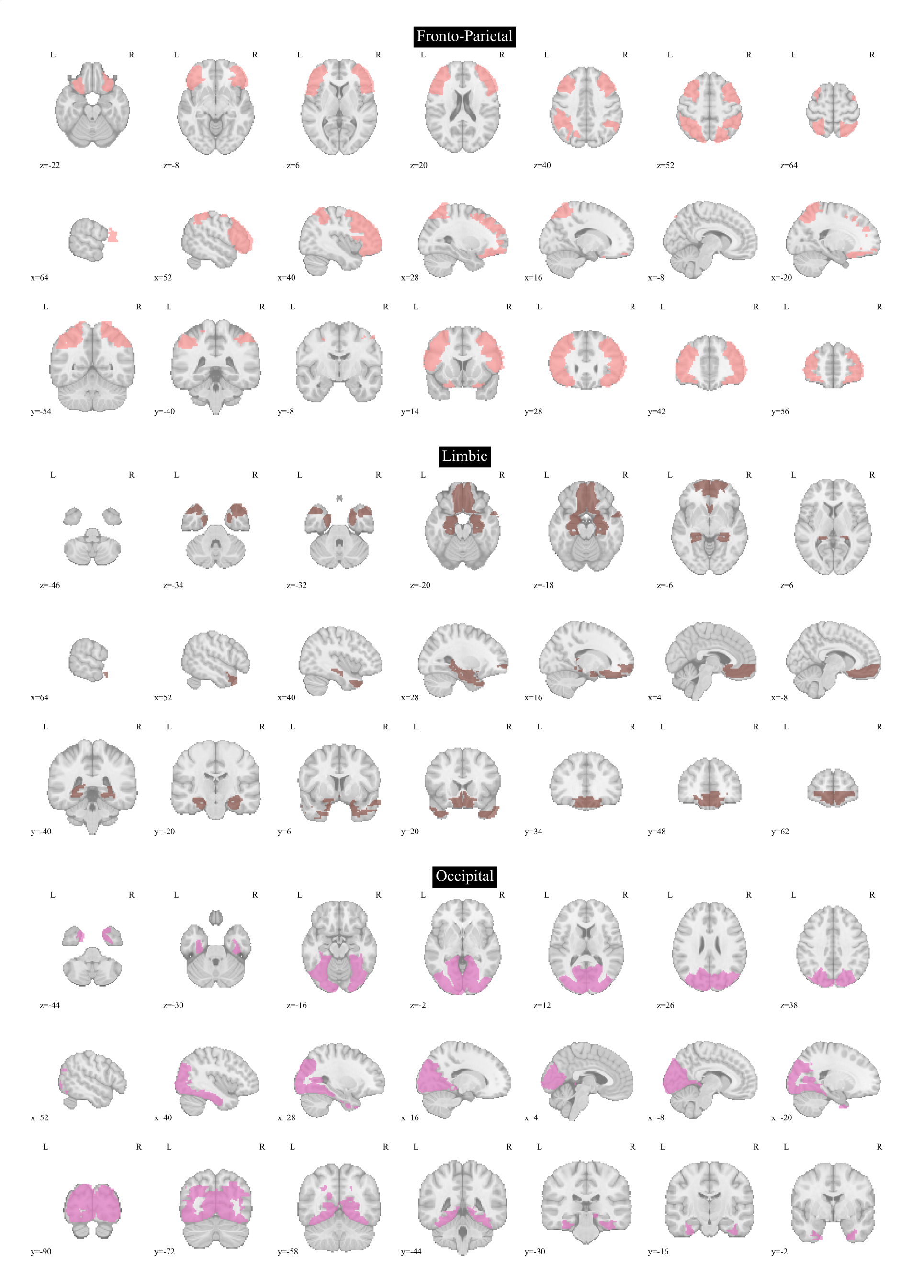

Figure S9. Resting state networks in the automated anatomic labelling (AAL) atlas. Top to bottom we see the Fronto-Parietal network, Limbic network, and Occipital Network.

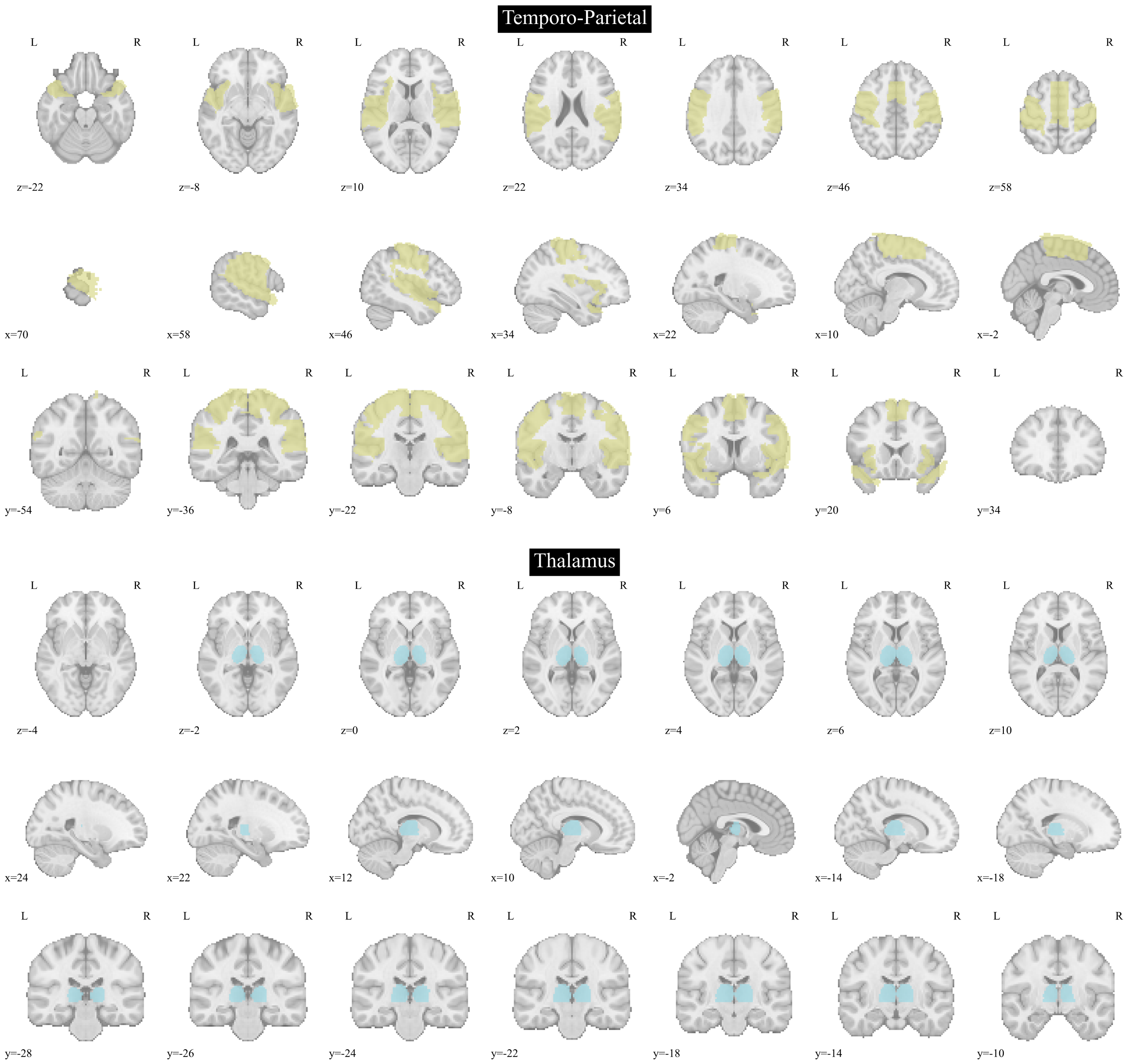

Figure S10. Resting state networks in the automated anatomic labelling (AAL) atlas. Top to bottom we see the Temporo-Parietal network and the Thalamus.

References

1. Ryan, M. *Riemannian statistical techniques with applications in fMRI*. Ph.D. thesis, The University of Adelaide (2023).

2. do Carmo, M. P. *Riemannian Geometry* (Birkhauser Boston Inc, 1992).

3. Pennec, X., Sommer, S. & Fletcher, T. *Riemannian Geometric Statistics in Medical Image Analysis* (Elsevier, 2019).
